# Chaperone-mediated autophagy directs a dual mechanism to balance premature senescence and senolysis to prevent intervertebral disc degeneration

**DOI:** 10.1038/s41413-025-00441-0

**Published:** 2025-06-12

**Authors:** Zhangrong Cheng, Haiyang Gao, Pengzhi Shi, Anran Zhang, Xianglong Chen, Yuhang Chen, Weikang Gan, Kangcheng Zhao, Shuai Li, Cao Yang, Yukun Zhang

**Affiliations:** Department of Orthopedics, Union Hospital, Tongji Medical College, Huazhong University of Science and Technology, Wuhan, Hubei China

**Keywords:** Pathogenesis, Bone

## Abstract

Intervertebral disc degeneration (IDD) is a progressive and dynamic process in which the senescence-associated secretory phenotype (SASP) of nucleus pulposus cells (NPC) plays a significant role. While impaired chaperone-mediated autophagy (CMA) has been associated with inflammation and cellular senescence, its specific involvement in the self-perpetuating feedback loop of NPC senescence remains poorly understood. Through LAMP2A knockout in NPC, we identified a significant upregulation of DYRK1A, a core mediator of premature senescence in Down syndrome. Subsequent validation established DYRK1A as the critical driver of premature senescence in CMA-deficient NPC. Combinatorial transcription factor analysis revealed that under IL1B stimulation or CMA inhibition, elevated DYRK1A promoted FOXC1 phosphorylation and nuclear translocation, initiating transcriptional activation of cell cycle arrest. Intriguingly, CMA impairment concurrently enhanced glutamine metabolic flux in senescent NPC, thereby augmenting their survival fitness. Transcriptomic profiling demonstrated that CMA reactivation in senescent NPC facilitated fate transition from senescence to apoptosis, mediated by decreased glutamine flux via GLUL degradation. Therefore, CMA exerts protective effects against IDD by maintaining equilibrium between premature senescence and senolysis. This study elucidates CMA’s regulatory role in SASP-mediated senescence amplification circuits, providing novel therapeutic insights for IDD and other age-related pathologies.

## Introduction

Tissue and organ aging is a complex, dynamic process. With the progressive shortening and severe erosion of telomeres, cells will enter a terminal growth arrest state, a process known as replicative senescence (RS). However, in more cases, cells face various external stresses and enter premature senescence in advance, also known as stress-induced premature senescence (SIPS).^1^ This type of senescence is essentially indistinguishable from RS-like phenotypes (including stable growth arrest). As an irreversible cell cycle arrest state induced by a variety of sublethal stress factors (such as oxidative stress, mechanical stress, and inflammation), the role of SIPS in bone and joint degenerative diseases has received increasing attention.^2,3^ Under pathological conditions, if senescent cells are not cleared in time, they will not only interfere with the normal function of tissues and organs, but also mediate the bystander senescent effect through the senescence-associated secretory phenotype (SASP), forming a senescence self-amplification feedback loop, thereby exacerbating the aging process of tissues and organs.^4^ SASP inflammatory factors represented by the IL1 family are the key to senescent cells inducing senescence of adjacent normal cells.^5^ Senescent cells are widely present in various organs and systems, becoming a potential factor in causing a variety of bone and joint degenerative diseases, such as osteoarthritis,^6^ intervertebral disc degeneration (IDD),^7^ osteoporosis,^8^ etc. At the same time, cells entering the senescent state also show reprogramming of pathways such as glutamine (Gln) metabolism to enhance their anti-apoptosis and survival adaptability,^9^ which makes senolytic treatment based on the strategy of clearing senescent cells face challenges.

IDD is a common, chronic, degenerative spinal disease that often causes lower back pain and disability in patients. Senescence and dysfunction of nucleus pulposus cells (NPC) are important causes of accelerated IDD.^10^ Compared with healthy disc tissue, the proportion of SA-β-gal-positive senescent cells and the expression levels of senescence-related markers such as TP53/P53, CDKN1A/P21, and CDKN2A/P16 in degenerated discs were significantly increased.^11^ In addition, NPC acquire SASP after senescence and release inflammatory factors such as IL1B, thereby promoting further aging and degeneration of the disc.^12^ In recent years, the clinical presentation of IDD has increasingly affected younger populations.^13^ Emerging research underscores that SIPS represents a pivotal amplifying factor in the early stages of IDD pathogenesis.^14^ A variety of stimuli, including oxidative stress, inflammatory mediators, and mechanical loading, are known to induce SIPS in NPC.^15^ These factors interact synergistically, accelerating NPC dysfunction and IDD progression,^16^ with disease onset as early as 20 years of age.^17^ Despite these insights, current therapeutic strategies for the early intervention and management of IDD remain limited and warrant further investigation.

Chaperone-mediated autophagy (CMA) is a selective lysosomal degradation pathway of specific substrates mediated by HSPA8 and LAMP2A (L2A).^18^ The basis of CMA’s selectivity is that all of its substrates contain a KFERQ-like pentapeptide motif that can be recognized by HSPA8.^19^ The expression levels of L2A are key determinants of CMA flux, with a notable age-related decline observed in both experimental models and human tissues.^20^ As a special mechanism evolved by mammalian cells, CMA can respond to various stimuli of SIPS, including inflammation,^21^ oxidative stress,^22^ DNA damage,^23^ and lipo-toxicity^24^. CMA not only promotes quality control by degrading damaged proteins, but also degrades undamaged proteins to terminate their function.^19^ In this way, CMA plays an important role in cell cycle, metabolism, growth, and senescence.^18^ CMA activity in many cells and tissues decreases with age,^25^ and reactivating CMA can delay the aging process of different tissues and organs.^26,27^ Under oxidative stress, DNA damage, and other conditions of SIPS, CMA exhibits an adaptive response by upregulating its activity to mitigate stress-induced damage and delay cellular senescence. For instance, CMA modulates the Keap1-Nrf2 pathway to confer resistance against oxidative stress-induced senescence.^22^ However, under inflammatory conditions, CMA activity is significantly suppressed, a phenomenon first described in Zhang’s study.^28^ This appears to involve cathepsin A-dependent L2A degradation and activation of the AKT signaling pathway. Subsequently, we also found that CMA attenuation was associated with TNF-induced SIPS in NPC.^21^ In particular, as inflammation induces a decline in CMA function, cells are more susceptible to oxidative stress, lipid stress, mitochondrial dysfunction, protein toxicity, etc., leading to further aggravation of the aging process of tissues and organs.^22,29,30^ Our previous studies have found that L2A was decreased in degenerated intervertebral discs, supporting CMA inhibition as a potential pathological factor in IDD,^21^ but its role needs to be further explored. It has been reported that CMA limits the generation and release of IL1B by degrading key components of inflammasomes.^31^ The specificity of CMA response in an inflammatory environment prompted us to further study the role of CMA in the IL1B-mediated senescence amplification feedback of NPC. This direction encompasses many currently unresolved questions, including the responsiveness of CMA activity of NPC to IL1B, the transcriptional regulatory programs controlled by CMA during normal NPC senescence, the consequences of CMA alterations on the survival of senescent NPC, and the potential of CMA to serve as a universal coordinator in the senescence amplification network.

Here, we investigate how CMA activity influences the SASP factor IL1B-induced senescence of NPC, and explore the effects of CMA activators on senescent NPC functionality and survival. Our findings reveal that CMA plays a critical role in maintaining the youthful state of rat intervertebral discs. CMA activation not only inhibited IL1B- or needle puncture-induced IDD, but also CMA dysfunction itself served as a stress condition promoting SIPS of NPC. Mechanistically, through proteomic analysis of L2A knockout NPC, we identified DYRK1A (a biomarker associated with accelerated aging in Down syndrome) as a previously unrecognized mediator linking CMA impairment to cellular senescence. We observed CMA suppression across multiple senescent NPC models. Intriguingly, CMA inhibition was found not merely as a senescence marker, but sustained senescent NPC viability by facilitating glutamine metabolic adaptation. Conversely, CMA activation reduced glutamine flux through GLUL degradation and induced apoptosis in senescent NPC. We propose that activated CMA functions as a biological rheostat, preventing premature NPC senescence while enabling selective clearance of senescent cells. This dual regulatory mechanism provides novel insights into the therapeutic potential of CMA activators for anti-aging interventions within the complex microenvironment of degenerating discs containing both normal and senescent NPC. Our findings extend the current understanding of CMA’s role from a passive aging-associated feature to an active modulator influencing senescence amplification loops, particularly in the context of IDD pathophysiology.

## Results

### CMA is essential for maintaining the youthful state of the nucleus pulposus

**Fig. 1:**
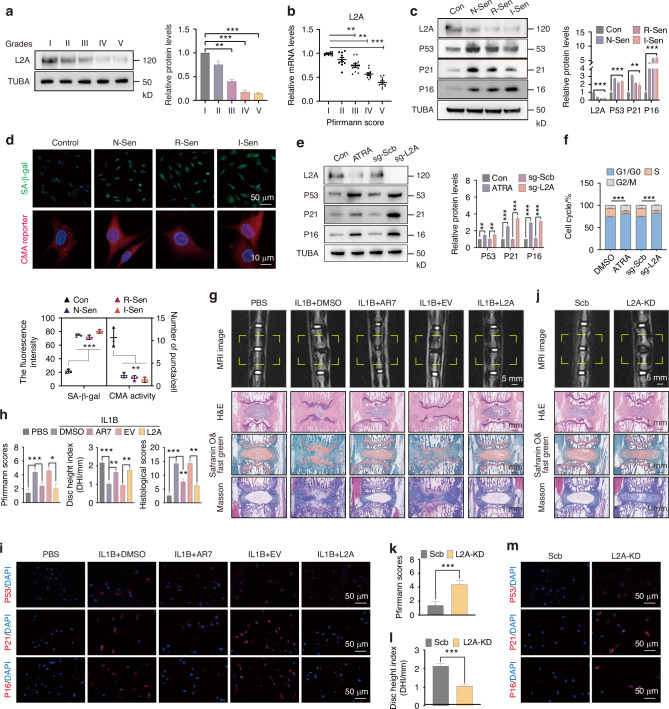
CMA is essential for maintaining the youthful state of the nucleus pulposus. **a**, **b** The protein and mRNA levels of L2A (LAMP2A) in human intervertebral discs with different degeneration grades. **c** The protein levels of L2A, P53, P21, and P16 in different senescent NPC models, N-Sen (Nutlin-3a-induced senescence), R-Sen (Replicative senescence, passage 9), and I-Sen (IL1B-induced senescence). **d** Immunofluorescence shows the levels of SA-β-gal and CMA activity in different senescent NPC models. CMA activity is represented by the average number of red spots per cell. **e** The protein levels of L2A, P53, P21, and P16 in NPC treated with ATRA or sg-L2A transfection. **f** Flow cytometry results of the cell cycle of each group. **g** The effect of CMA activation on IL1B-induced IDD. MRI imaging and histological staining were performed after sampling. **h** The grade of IDD in each group was evaluated by Pfirrmann scores, DHI, and histological scores, as described before.^77^ **i** Immunofluorescence showing levels of P53, P21, and P16 in the rat discs. **j**–**l** The effect of L2A-KD on rat IDD, evaluated by Pfirrmann grading and DHI. **m** The effect of L2A-KD on in vivo levels of P53, P21, and P16. All graphs show the mean ± SEM of at least three independent experiments. *P <0.05, **P <0.01, ***P <0.001

To investigate the CMA levels during IDD, we detected the expression of LAMP2A (L2A) in degenerated human disc samples. The results showed that the protein and mRNA levels of L2A decreased with the severity of IDD (Fig. 1a, b). We used MDM2 inhibitor (nutlin-3a, N-Sen) and inflammatory factor (IL1B, I-Sen) to induce NPC senescence, and used replicative cell senescence (Passage 9, R-Sen) as a positive control. The results showed that the expression levels of L2A were significantly decreased in N-Sen, I-Sen, and R-Sen, accompanied by increased expression of senescence-related proteins TP53/P53, CDKN1A/P21, and CDKN2A/P16 (Fig. 1c), indicating that CMA is inhibited in senescent NPC. At the same time, there was an increase in SA-β-gal activity and a decrease in CMA activity in the NPC of N-Sen, I-Sen and R-Sen groups (Fig. 1d). We further explored the role of CMA in maintaining the youthful state of NPC, and found the expression levels of senescence-related proteins (P53, P21 and P16) were increased in NPC treated with CMA inhibitor ATRA or sg-L2A transfection (Fig. 1e). At the same time, CMA inhibition promoted cell cycle arrest, as manifested by a decrease in the proportion of NPC in S phase (Fig. 1f). We further studied the effect of CMA activation on IDD induced by IL1B in the caudal disc of rats. MRI and histological staining showed that local injection of IL1B after 8 weeks induced disc degeneration (Fig. 1g, h). By co-injecting CMA activator AR7 or L2A overexpression lentivirus, the IDD degree was alleviated, represented by the decreased Pfirrmann scores, restored disc height (DHI), and reduced histological scores (Fig. 1g, h). Through the fluorescence intensity analysis of senescence-related proteins (P53, P21, and P16), we found that CMA activation improved NPC senescence induced by IL1B (Fig. 1i, S1a). In addition, local injection of AR7 or L2A lentivirus can also exert a protective effect in the rat IDD model induced by needle puncture (Fig. S1b). Further, we explored the effect of L2A knockdown (L2A-KD) on the progression of IDD. The results showed that local injection of sh-L2A lentivirus for 8 weeks induced IDD of rats, manifested by increased Pfirrmann and histological scores, and decreased DHI (Fig. 1j–l, S1c). Tissue immunofluorescence showed that the expression levels of P53, P21, and P16 in intervertebral disc cells were significantly increased after L2A-KD (Fig. 1m, S1d). In conclusion, these results suggest that CMA activity is crucial for maintaining the youthful state of the intervertebral disc.

### Elevated DYRK1A drives senescence in CMA-downregulated NPC

**Fig. 2:**
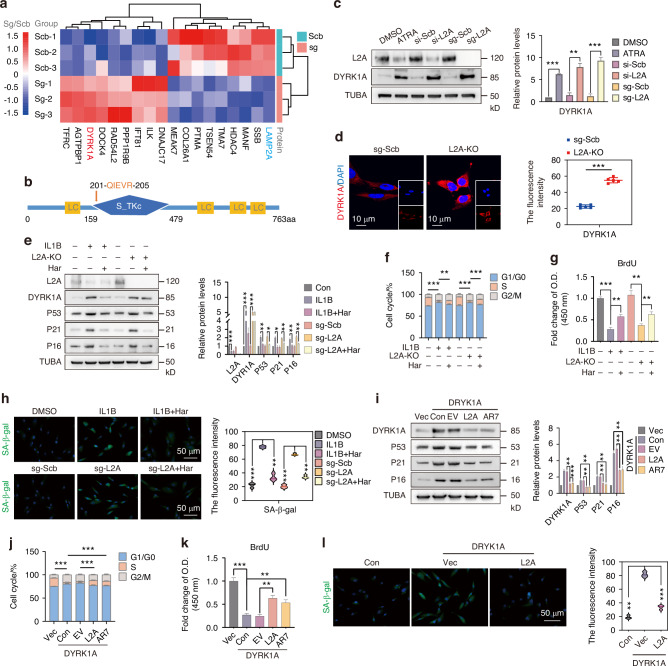
Elevated DYRK1A drives premature senescence in CMA-downregulated NPC. **a** TMT-MS analysis showing that DYRK1A levels were increased in NPC after L2A knockout. **b** Schematic diagram of the sequence of DYRK1A. **c** The protein level of DYRK1A in NPC treated with ATRA, si-L2A, or sg-L2A. **d** Immunofluorescence shows the level of DYRK1A in NPC after L2A knockout. **e** Immunoblotting shows the levels of L2A, DYRK1A, P53, P21, and P16 in NPC treated as indicated. NPC were treated with IL1B (10 ng/mL, 48 h) and Har (Harmine, 20 μg/mL, 48 h). **f** Flow cytometry showing the cell cycle of NPC treated as indicated. The proportion of NPC in the S phase was used for comparison. **g** Relative BrdU levels of NPC treated as indicated, measured at 450 nm. **h** SA-β-gal fluorescence levels detected by Fluorescein diβ-D-galactopyranoside (FDG) in NPC treated as indicated. **i** Western blot showing the protein levels of DYRK1A and senescence markers in NPC of each group. **j** Flow cytometry showing the cell cycle changes of NPC treated as indicated. **k** Relative BrdU levels of NPC treated as indicated. **l** Immunofluorescence showing SA-β-gal levels in NPC after DYRK1A and L2A overexpression. All graphs show the mean ± SEM of at least three independent experiments. *P <0.05, **P <0.01, ***P <0.001

To explore the mechanism by which CMA inhibition drives NPC senescence, we silenced CMA function using sg-L2A and performed TMT-labeled mass spectrometry analysis. The construction of sg-L2A targets a specific sequence of L2A without affecting the gene expression of other LAMP2 isoforms (Fig. S1e, f). Mass spectrometry screening results showed that DYRK1A, a pathogenic gene for human trisomy 21 Down syndrome, was significantly increased after L2A-KO (Fig. 2a). The gene encoding DYRK1A is located in the critical region for Down syndrome on human chromosome 21. The protein is 763 amino acids long and contains the classic CMA targeting sequence _201_QIEVR_205_ (Fig. 2b). DYRK1A levels were increased in several CMA inhibition conditions (Fig. 2c). However, the mRNA level of DYRK1A was not affected (Fig. S1g). Immunofluorescence showed that the expression level of DYRK1A was increased after L2A-KO (Fig. 2d). After IL1B treatment, the expression of L2A was inhibited in NPC, accompanied by increased senescence-related proteins (P53, P21, and P16). DYRK1A levels were also increased after IL1B treatment (Fig. 2e), but they were decreased in I-Sen NPC after AR7 treatment or L2A overexpression (Fig. S1h). Notably, the use of DYRK1A inhibitor Harmine (Har) rescued NPC senescence caused by IL1B or L2A-KO (Fig. 2e). At the same time, cell cycle arrest caused by IL1B or L2A-KO was also inhibited by Harmine (Fig. 2f). The detection of BrdU showed that NPC proliferation was inhibited by IL1B or L2A-KO, while Harmine partially restored this process (Fig. 2g). We found that the increased expression of SA-β-gal induced by IL1B or sg-L2A transfection was reversed by Harmine (Fig. 2h). RT-qPCR results showed that IL1B or L2A-KO increased the mRNA levels of senescence-related proteins (P53, P21, P16) and SASP inflammatory factors (IL1B, IL6, IL-8), while the addition of Harmine inhibited these increases (Fig. S2a, b). By stably transfecting DYRK1A, we found that the expression of senescence-related proteins (P53, P21, and P16) in NPC was increased, but this effect was inhibited after co-transfection of L2A or AR7 co-treatment (Fig. 2i). At the same time, overexpression of DYRK1A also led to cell cycle arrest in NPC. Overexpression of L2A or co-treatment of AR7 can reverse the cell cycle arrest caused by DYRK1A overexpression (Fig. 2j, k). In addition, L2A overexpression could alleviate the activation of SA-β-gal in NPC caused by DYRK1A overexpression (Fig. 2l). Similarly, DYRK1A overexpression promoted the mRNA levels of senescence-related proteins (P53, P21, P16) and SASP inflammatory factors (IL1B, IL6, IL-8), which were reversed by co-overexpression of L2A or AR7 co-treatment (Fig. S2c, d). In rat models, local overexpression of DYRK1A also accelerates IDD progression, while L2A co-expression inhibits this process (Fig. S2e, f). In rat intervertebral disc tissue, lentiviral-mediated overexpression of DYRK1A upregulated the expression of senescence-associated proteins, including P53, P21 and P16, while overexpression of L2A ameliorated this aging phenotype (Fig. S2g, h). The NF-κB signaling pathway is a known pathway regulating NPC senescence and SASP activation in IDD.^32^ We wondered whether the mechanism by which CMA regulates NPC senescence through DYRK1A involves this canonical senescence pathway. Our results showed a significant decrease in the level of IKBA protein in NPC after DYRK1A overexpression (Fig. S3a). Concomitantly, immunofluorescence assays revealed an increase in RelA nuclear translocation (Fig. S3b), indicating that the NF-κB pathway was activated by DYRK1A overexpression. Notably, L2A overexpression rescued the DYRK1A-induced decrease in IKBA levels and inhibited RelA nuclear translocation, thereby suppressing NF-κB pathway activation (Fig. S3a, b). Collectively, our findings demonstrate that the senescence of NPC induced by CMA inhibition is associated with upregulation of DYRK1A, and DYRK1A-induced senescence of NPC can be ameliorated through either pharmacological inhibition of DYRK1A or activation of CMA.

### DYRK1A is a bona fide substrate of CMA in NPC

**Fig. 3:**
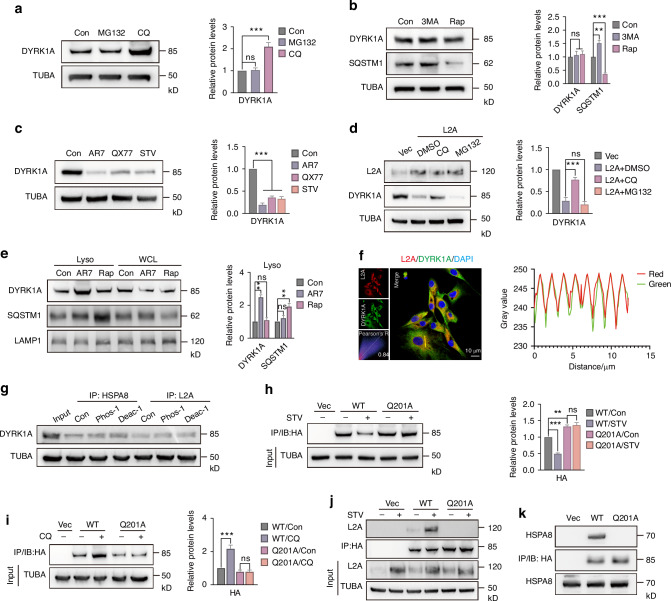
DYRK1A is a bona fide substrate of CMA in NPC. **a** Western blot showing DYRK1A levels after MG132 (5 μmol/L) or Chloroquine (CQ, 20 μmol/L) treatment for 12 h. **b** Immunoblots showing DYRK1A and SQSTM1 levels in NPC treated with 3MA (5 mmol/L) or Rap (Rapamycin, 1 μmol/L) for 12 h. **c** Immunoblots showing DYRK1A levels in NPC treated with AR7 (50 μmol/L), QX77 (25 μmol/L), and starvation for 24 h. **d** Western blot showing DYRK1A levels in NPC after L2A overexpression or combined with CQ/MG132. **e** Western blot shows the levels of DYRK1A and SQSTM1 in lysosomes or cytoplasm of NPC treated with AR7 or Rap. **f** Immunofluorescence showing that L2A and DYRK1A colocalized in NPC. **g** Immunoprecipitation showing the effect of phosphatase inhibitors and deacetylase inhibitors on the levels of DYRK1A bound to HSPA8 or L2A in NPC. **h** Western blot showing the effect of starvation on HA levels in NPC transfected with WT or Q201A mutant HA-DYRK1A plasmids. **i** Immunoblotting shows the effect of CQ (20 μmol/L) on HA levels in NPC transfected with WT or Q201A mutant HA-DYRK1A plasmids. **j** Co-immunoprecipitation analysis shows the effect of starvation on the level of L2A bound by HA-DYRK1A in NPC transfected with WT or Q201A. **k** Co-immunoprecipitation results showing the level of HA-bound HSPA8 in NPC transfected with WT or Q201A mutant HA-DYRK1A. All graphs show the mean ± SEM of at least three independent experiments. *P <0.05, **P <0.01, ***P <0.001, ns indicates not significant

To further explore the degradation pathway of DYRK1A, we treated NPC with MG132 and Chloroquine (CQ). The results showed that the level of DYRK1A did not change in the MG132-treated group, while it was increased significantly after CQ inhibited lysosomal enzymes (Fig. 3a). At the same time, the level of DYRK1A in NPC was gradually increased at 4 h, 8 h and 12 h after CQ administration (Fig. S3c). Further, we treated NPC with the macroautophagy inhibitor 3-methyladenine (3MA) and its activator rapamycin (Rap), respectively. The results showed that the protein level of DYRK1A was not affected, indicating that DYRK1A is not regulated by macroautophagy (Fig. 3b). However, the level of DYRK1A was significantly decreased after NPC were treated with several CMA activation conditions, including AR7, QX77, and starvation treatments (Fig. 3c). Further experiments showed that only CQ, but not MG132, could rescue the reduction of DYRK1A induced by L2A overexpression (Fig. 3d). Next, we studied the level of DYRK1A in lysosomes. We treated NPC with AR7 or Rap and isolated their lysosomes, and found that DYRK1A was significantly increased in the lysosomes of NPC treated with AR7, while it was not affected in the Rap treatment group (Fig. 3e). As a positive control for macroautophagy, SQSTM1 in lysosomes was increased after Rap treatment, but was not significantly affected by AR7 (Fig. 3e). Another important feature of CMA substrates is their structural association with CMA elements. We performed colocalization analysis on the fluorescence images of DYRK1A and L2A, and found that the two had significant overlap (Fig. 3f). In the protein sequence of DYRK1A, we found not only the classic KFERQ-like sequence _201_QIEVR_205_ (Fig. 2b), but also several other hypothetical sites that meet the conditions after phosphorylation or acetylation. After treatment with phosphorylase inhibitors and deacetylase inhibitors in NPC, we found that the binding of DYRK1A to HSPA8 or L2A was not affected, indicating that the CMA target sequence of DYRK1A was not regulated by these two post-translational modifications (Fig. 3g). Q-A mutation is usually an effective way to disrupt the binding of CMA substrates and HSPA8.^33^ Further, we performed a structural mutation of Q201A on _201_QIEVR_205_ of DYRK1A and explored the reactivity of HA-Q201A under starvation conditions. Immunoprecipitation results showed that starvation reduced the level of HA-DYRK1A in the WT group, while the HA-DYRK1A level in the Q201A group was not affected (Fig. 3h). Similarly, in response to CQ, the WT group, but not the Q201A mutant group, showed blocked degradation of HA-DYRK1A (Fig. 3i). There was co-immunoprecipitation of L2A and DYRK1A in the WT group, and this binding was enhanced after starvation treatment (Fig. 3j). However, no binding of HA-DYRK1A to L2A was shown in the mutant group (Fig. 3j). In the WT group, but not in the Q201A mutant group, we detected co-immunoprecipitation of DYRK1A and HSPA8 (Fig. 3k, S3d). In summary, the above findings indicate that DYRK1A is a bona fide CMA substrate in NPC.

### FOXC1 phosphorylated by DYRK1A enters the nucleus and initiates cell cycle arrest and premature senescence of NPC

**Fig. 4:**
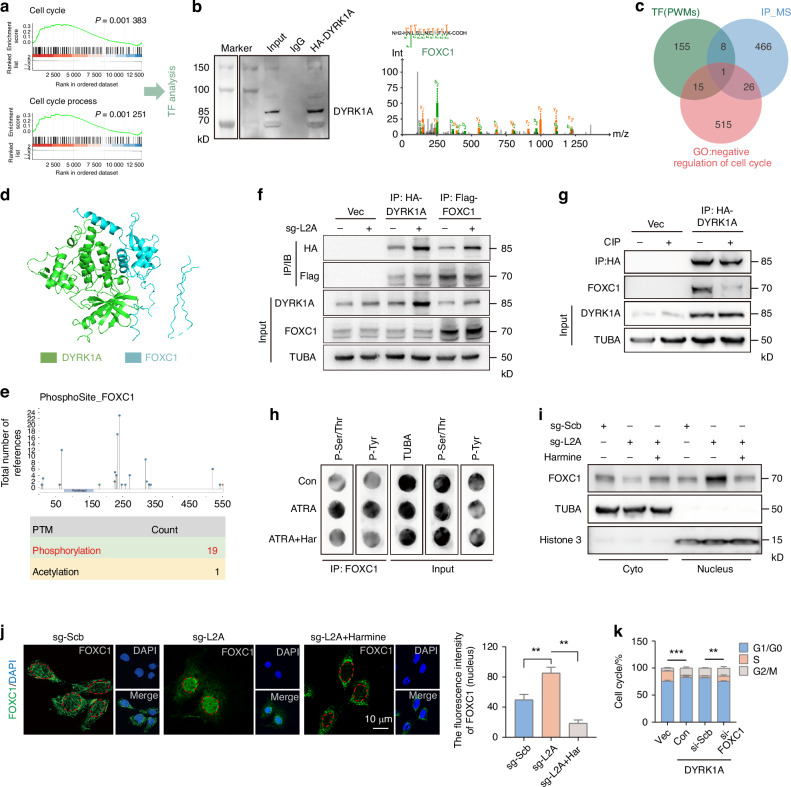
FOXC1 phosphorylated by DYRK1A enters the nucleus and initiates cell cycle arrest and premature senescence of NPC. **a** GSEA analysis showing that cell cycle pathways were significantly enriched after DYRK1A overexpression in NPC. **b** IP-MS analysis showed that FOXC1 was enriched in the immunoprecipitated complexes of DYRK1A. **c** Cross-analysis of TF predicted by transcriptomic data of DYRK1A overexpression, IP-MS results, and GO gene sets that negatively regulate the cell cycle revealed that FOXC1 was the potential target. **d** Protein binding analysis of DYRK1A and FOXC1 by ZDOCK.^78^ **e** PTM analysis of phosphorylation in FOXC1. **f** Immunoprecipitation blot showing that DYRK1A and FOXC1 were bound. **g** The effect of phosphatase CIP on the levels of DYRK1A-bound FOXC1. **h** Dot blots show the effect of ATRA and Har on the phosphorylation of FOXC1. **i** Immunoblots show the effect of L2A knockout or combined with Har on the nuclear and cytoplasmic distribution of FOXC1 in NPC. **j** Immunofluorescence showing the effects of sg-L2A and Har on the nuclear and cytoplasmic distribution of FOXC1 in NPC. **k** The effect of DYRK1A overexpression or combined with FOXC1 knockdown on the cell cycle of NPC. All graphs show the mean ± SEM of at least three independent experiments. *P <0.05, **P <0.01, ***P <0.001

We found that DYRK1A mediated NPC senescence under CMA inhibition. After overexpression of DYRK1A in NPC, GSEA analysis showed significant changes in KEGG pathways related to the cell cycle (Fig. 4a). The regulation of cell growth, development, and senescence by DYRK1A is primarily mediated through phosphorylation modification of transcription factors. To identify DYRK1A-associated proteins, we performed immunoprecipitation (IP) coupled with mass spectrometry (MS) analysis (Fig. 4b), followed by stringent screening to identify significant transcription factors (TF). Through integrated analysis of the IP-MS data, transcriptome data from NPC cells overexpressing DYRK1A, and the GO gene set associated with negative cell cycle regulation, we conclusively identified FOXC1 as the sole transcription factor meeting all the predefined criteria (Fig. 4c). From the aspect of protein structure, we performed molecular docking simulation and found that DYRK1A and FOXC1 have a tight structural binding (Fig. 4d). Meanwhile, PTM analysis of FOXC1 showed that phosphorylation was its most important post-translational modification, with up to 19 phosphorylation sites (>90%) (Fig. 4e). From the perspective of protein interaction, we examined that DYRK1A and FOXC1 were co-immunoprecipitated (Fig. 4f). Although DYRK1A level was increased after sg-L2A transfection, the overall level of FOXC1 was not affected significantly (Fig. 4f). After the phosphorylation binding was destroyed with phosphatase CIP, we found that DYRK1A-bound FOXC1 was significantly reduced (Fig. 4g). Next, we used dot blot to detect changes in FOXC1 phosphorylation levels. Immunoprecipitation showed that FOXC1 bound serine/threonine and tyrosine, and the phosphorylation level of FOXC1 also changed under CMA and DYRK1A inhibitors. After treatment with the CMA inhibitor ATRA, the total phosphorylation levels of FOXC1 were increased (Fig. 4h). After inhibiting DYRK1A activity with Harmine (Har), the overall phosphorylation levels of FOXC1 were decreased (Fig. 4h). This indicated that CMA-DYRK1A axis affected the phosphorylation level of FOXC1, which could subsequently affect the function and localization of FOXC1. Next, we performed nuclear-cytoplasmic separation to detect FOXC1 levels under L2A-KO or Harmine treatment. After L2A-KO, the level of FOXC1 in the nucleus was increased significantly, while the cytoplasmic level was decreased accordingly. It is worth noting that the combined treatment with Harmine reversed this effect (Fig. 4i). Cell immunofluorescence also showed that after L2A-KO, the nuclear translocation of FOXC1 was enhanced, while it was significantly inhibited after the function of DYRK1A was inhibited by Harmine, even below the initial level (Fig. 4j). Therefore, the phosphorylation degree of FOXC1 by DYRK1A could regulate the nuclear localization of FOXC1.

Further, we studied the effects of FOXC1 under the regulation of DYRK1A on the cell cycle and senescence of NPC. After DYRK1A overexpression, the mRNA levels of cell cycle arrest markers (MKI67, PCNA, and p27) and cell senescence markers (P53, P21 and P16) in NPC were increased, while knockdown of FOXC1 rescued these changes (Fig. S3e, f). Similarly, knockdown of FOXC1 also inhibited the increase in SA-β-gal activity induced by DYRK1A overexpression (Fig. S3g, h). We utilized the infrared fluorescent tracker of SA-β-gal to track in vivo senescent cells in a live rat model. We found that DYRK1A overexpression for one month resulted in a significant promotion of SA-β-gal activation in the intervertebral disc of rats (Fig. S3i). Furthermore, local L2A overexpression or FOXC1 knockdown via lentivirus could reduce the levels of DYRK1A-induced SA-β-gal (Fig. S3i). Flow cytometric analysis revealed that si-FOXC1 rescued the cell cycle arrest of NPC induced by DYRK1A overexpression or L2A knockdown (Fig. 4k, S3j). Immunoblotting further demonstrated that DYRK1A overexpression or L2A knockdown significantly increased the expression of proteins associated with cellular senescence, and these effects were offset by FOXC1 gene silencing (Fig. S3k–m). In addition, while DYRK1A overexpression promoted the activation of NF-κB signaling, the canonical senescence pathway in IDD, as evidenced by increased nuclear translocation of RelA and reduced IKBA levels, FOXC1 knockdown was able to mitigate the effects of DYRK1A and suppress the activation of the NF-κB pathway (Figs. S3n, S4a). Lentiviral-mediated in vivo knockdown of FOXC1 effectively ameliorated rat IDD induced by DYRK1A overexpression, as evidenced by elevated disc height (DHI) and improved Pfirrmann and histological scores (Fig. S4b, c). Moreover, tissue immunofluorescence analysis demonstrated that FOXC1 knockdown significantly reduced the expression levels of DYRK1A-induced senescence-associated proteins, including P53, P21 and P16 (Fig. S4d, e). In summary, the findings indicate that the phosphorylation-dependent activation and nuclear translocation of FOXC1 represent the molecular mechanism underlying DYRK1A-induced NPC senescence. The DYRK1A-FOXC1 axis serves as a critical regulatory target for CMA in modulating the transcriptional processes associated with NPC senescence.

### CMA induces a fate switch of senescent NPC from cellular senescence to apoptosis

**Fig. 5:**
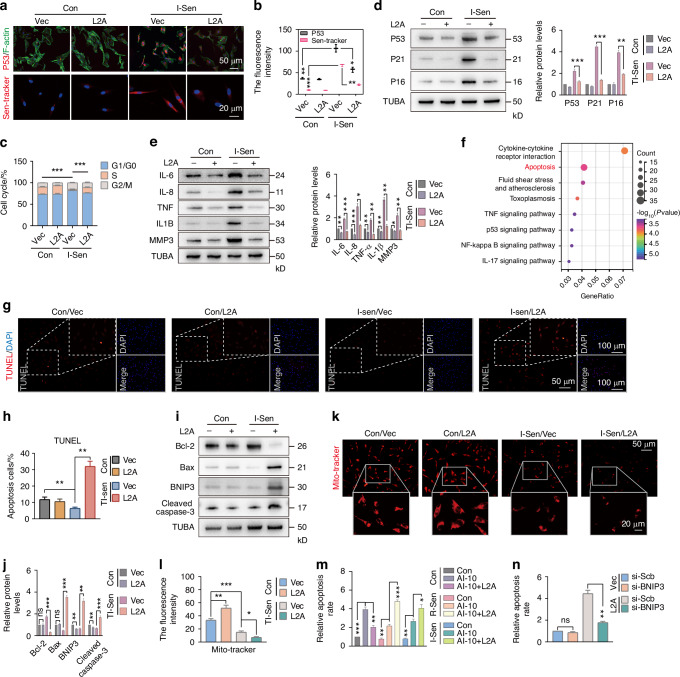
CMA induces a fate switch of senescent NPC from cellular senescence to apoptosis. **a**, **b** Immunofluorescence showing the effect of L2A overexpression on P53 and senescence levels in I-Sen NPC. **c** The effect of L2A overexpression on the cell cycle of NPC in the control or I-Sen group. **d** Western blot showing the effect of L2A overexpression on the levels of senescence-related proteins (P53, P21, P16) in I-Sen NPC. **e** Western blot showing the effect of L2A overexpression on the levels of SASP inflammatory factors (IL6, IL-8, IL1B, TNF) and MMP3 in I-Sen NPC. **f** After treatment with CMA activator AR7 in I-Sen NPC, RNA-seq revealed that apoptosis-related pathways were significantly enriched. **g**, **h** TUNEL staining shows the apoptosis level in I-Sen NPC after L2A overexpression. **i**, **j** Immunoblotting showing the effect of L2A overexpression on the expression of apoptosis-related proteins in I-Sen NPC. **k**, **l** The effect of L2A overexpression on mitochondrial membrane potential in NPC of each group. **m** The apoptosis levels in normal and senescent NPC (R-Sen and I-Sen) treated with AI-10 or combined with L2A overexpression. **n** The effect of L2A overexpression or combined with BNIP3 knockdown on the apoptosis of senescent NPC. All graphs show the mean ± SEM of at least three independent experiments. *P <0.05, **P <0.01, ***P <0.001, ns indicates not significant

Given that senescent NPC play a key role in the accelerated degeneration of IVD,^34,35^ we next investigated how restoration of CMA activity affects IL1B-induced senescent NPC, I-Sen. Our findings revealed that overexpression of L2A in I-Sen NPC ameliorated the senescence phenotype, as demonstrated by decreased P53 fluorescence intensity and reduced SA-β-gal activity in live cells (Fig. 5a, b). RT-qPCR analysis further indicated that L2A overexpression significantly reduced the mRNA levels of key senescence markers, including P53, P21 and P16 (Fig. S5a), as well as the mRNA levels of SASP-related inflammatory factors, such as IL1B, IL6, and IL-8 (Fig. S5b). Additionally, overexpression of L2A not only improved cell cycle progression in I-Sen NPC (Fig. 5c) but also decreased the protein expression levels of P53, P21, and P16 in I-Sen NPC (Fig. 5d) and alleviated the expression of SASP factors (Fig. 5e). Flow cytometry analysis suggested that overexpression of L2A significantly reduced the expression level of P16 in I-Sen NPC (Fig. S5c). These findings collectively suggest that activation of CMA can effectively alleviate the senescent phenotype in I-Sen NPC.

To investigate the functional impact and underlying mechanism of restoring CMA activity in I-Sen, we conducted transcriptome-wide sequencing analysis of I-Sen following treatment with CMA activation. Pathway enrichment analysis revealed that CMA activation significantly enhanced apoptosis in I-Sen NPC (Fig. 5f). It is well-established that senescent cells undergo metabolic rewiring and develop resistance to apoptosis.^36^ Strategies targeting the elimination of senescent cells (senolytics) have demonstrated efficacy in ameliorating the senescent phenotype in degenerated tissues and organs. Notably, CMA has been reported to exhibit a dual role in regulating programmed cell death in non-senescent cells, such as ferroptosis, depending on cell type and context.^37,38^ Based on these observations, we propose that the improvement of the overall senescent phenotype in NPC mediated by CMA activation may involve the selective clearance of senescent NPC. Furthermore, CMA activation may influence the survival adaptability of senescent NPC and modulate their susceptibility to apoptosis. Subsequently, we demonstrated through TUNEL staining and immunoblotting that L2A overexpression-induced apoptosis in I-Sen NPC, as evidenced by an increased Bax/Bcl-2 ratio and enhanced expression of BNIP3 and cleaved caspase 3 (Fig. 5g–j), Furthermore, flow cytometry analysis showed that L2A overexpression significantly induced apoptosis in R-Sen and N-Sen NPC (Fig. S5d–i). Notably, the basal apoptosis level of I-Sen NPC was lower than that of normal NPC, potentially due to the elevated Bcl-2-mediated resistance to apoptosis in senescent NPC (Fig. 5g–j). Apoptosis is usually divided into intrinsic (mitochondrial) and extrinsic pathways.^39^ Mitochondrial staining analysis suggested that L2A overexpression might trigger intrinsic apoptosis through mitochondrial membrane potential damage (Fig. 5k, l).

To further investigate the role of L2A overexpression in apoptosis susceptibility under AI-10, a well-known apoptosis inducer, we performed flow cytometry analysis. The results indicated that AI-10 induced apoptosis in both R-Sen and I-Sen NPC groups. However, the apoptotic response to AI-10 was weaker in senescent NPC compared to normal NPC (Fig. 5m). Importantly, L2A overexpression enhanced the susceptibility of senescent NPC to AI-10-induced apoptosis, while reducing the apoptotic response in normal NPC (Fig. 5m). Additionally, L2A overexpression directly induced apoptosis in I-Sen NPC, an effect that was alleviated upon BNIP3 knockdown via si-BNIP3 (Fig. 5n). These findings suggest that normal and senescent NPC exhibit distinct responsiveness to CMA activation. While CMA activation exerts an apoptosis-protective role in normal NPC, it promotes apoptosis in senescent NPC. Interestingly, apoptosis rescue experiments revealed that BNIP3 knockdown not only inhibited L2A-induced apoptosis in I-Sen NPC (Fig. 5n) but also counteracted the anti-senescence effect of L2A. This was evidenced by the increased protein and mRNA levels of senescence-related proteins (P53, P21, P16) and SASP inflammatory factors (IL1B, IL6, and IL-8) (Fig. S6a–c). We further performed in vivo experiments to investigate the effects of L2A overexpression and BNIP3 knockdown on intervertebral disc senescence. Utilizing an infrared fluorescent probe targeting SA-β-gal, we tracked senescent NPC in vivo and observed that L2A overexpression significantly alleviated NPC senescence induced by IL1B injection. Conversely, sh-BNIP3 lentivirus abolished the senescence-protective effect of L2A (Fig. S6d). At the cellular level, flow cytometry was employed to quantify P16 expression in I-Sen NPC, while fluorescence-based assays were used to measure intracellular P53 levels and live-cell senescent signals (Fig. S6e, f). These findings demonstrated that BNIP3 knockdown inhibited the beneficial effects of L2A overexpression on mitigating NPC senescence, thereby underscoring the crucial role of CMA in regulating the balance between senescence and apoptosis in NPC. Collectively, CMA activation drives a shift in NPC fate from senescence to apoptosis, thereby facilitating the elimination of senescent cells and alleviating the overall aging process of the disc.

### Restoring CMA activity in senescent NPC inhibits glutamine synthesis, thereby promoting GLS1-dependent apoptosis

**Fig. 6:**
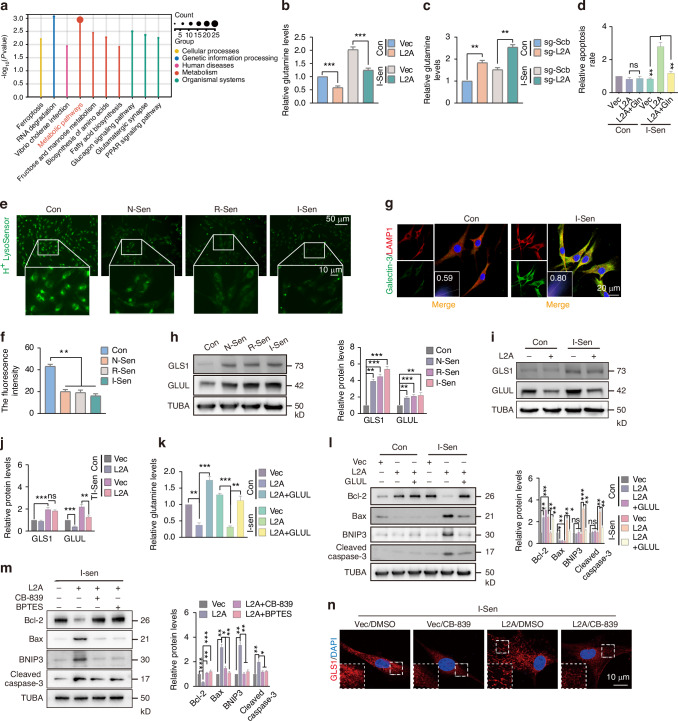
Restoring CMA activity in senescent NPC inhibits glutamine synthesis, thereby promoting GLS1-dependent apoptosis. **a** Proteomic analysis of L2A-KO NPC showing that cellular metabolism was enriched. **b** The glutamine levels in control or I-Sen NPC after L2A overexpression. **c** The effect of L2A knockout on glutamine levels. **d** The effect of L2A overexpression or combined with glutamine on the apoptosis level of control or I-Sen NPC. **e**, **f** The acidity of lysosomes in normal or senescent NPC. **g** The colocalization of the membrane damage marker Galectin-3 and LAMP1 in control or I-Sen NPC. **h** The protein levels of GLS1 and GLUL in normal or senescent NPC. **i**, **j** The effect of L2A overexpression on the expression of GLS1 and GLUL in normal and I-Sen NPC. **k** The effect of L2A or combined with GLUL overexpression on glutamine levels in I-Sen NPC. **l** Immunoblotting showing the effect of L2A or combined with GLUL overexpression on apoptosis in I-Sen NPC. **m** GLS1 inhibitors CB-839 and BPTES inhibited I-Sen NPC apoptosis after L2A overexpression. **n** The effect of L2A overexpression and CB-839 on GLS1 filaments in I-Sen NPC. All graphs show the mean ± SEM of at least three independent experiments. *P <0.05, **P <0.01, ***P <0.001, ns indicates not significant

The regulation of cell fate by CMA is often determined by the function of its target substrates. To investigate the mechanism underlying CMA activation-induced apoptosis of senescent NPC, we analyzed mass spectrometry data that identified potential CMA substrates, which showed that cellular metabolism pathways were the most significantly enriched following L2A-KO (Fig. 6a). This suggests that CMA may influence the function and survival of senescent NPC through metabolic regulation. Among the predicted CMA substrates identified in the list, GLUL emerged as a protein of particular interest (Fig. S7a). GLUL is a key enzyme required for intracellular glutamine synthesis. Notably, metabolic reprogramming is a hallmark of cellular senescence, especially the reprogrammed glutamine metabolism that provides potential targets for senolytics.^9,40^ Glutamine metabolism not only plays a role in cellular energy metabolism but also contributes to the survival adaptability of senescent cells through acid-based regulation.^9^ Through quantification of glutamine levels, we observed that senescent NPC exhibited significantly higher baseline glutamine levels compared to their normal counterparts. Importantly, both cell types displayed reduced glutamine levels following L2A overexpression (Fig. 6b). Conversely, L2A knockout led to an increase in glutamine levels (Fig. 6c). Given the critical role of glutamine flux in sustaining the survival of senescent cells,^9^ we investigated whether the CMA activation-dependent reduction of glutamine levels in senescent NPC influences their apoptotic response. Flow cytometry analysis revealed that L2A overexpression enhanced apoptosis in senescent NPC, an effect that was abolished by glutamine supplementation (Fig. 6d). Notably, normal NPC exhibited minimal changes in apoptosis regardless of CMA activation or glutamine addition, suggesting a differential sensitivity to glutamine metabolism between normal and senescent cells. This discrepancy may stem from the distinct metabolic requirements of these cell states. In senescent cells, lysosomal damage and H^+^ leakage are commonly observed, necessitating the neutralization of the intracellular acidic environment via glutamine metabolism to maintain survival.^41^ Consistent with this, we identified key features of lysosomal dysfunction in senescent NPC, including decreased lysosomal acidity (Fig. 6e, f) and extensive colocalization of the lysosomal membrane damage marker galectin-3 with LAMP1 (Fig. 6g).

Active glutamine metabolism is an important feature of senescent cells.^41^ In our study, comparative analysis revealed significant upregulation of glutamine synthetase GLUL and glutamine degrading enzyme GLS1 in various senescent models of NPC compared to normal NPC (Fig. 6h). Immunoblotting results demonstrated that GLUL levels, but not GLS1, were notably reduced in NPC following CMA activation (Fig. 6i, j). GLUL overexpression successfully mitigated the decrease in glutamine levels induced by L2A overexpression (Fig. 6k). Importantly, co-overexpression of GLUL rescued apoptosis of senescent NPC induced by L2A overexpression (Fig. 6l). Additionally, while DYRK1A has been shown to be upregulated in senescent NPC (Fig. 2e), knockdown of DYRK1A did not significantly influence the apoptosis of senescent NPC or the associated changes in glutamine levels (Fig. S7b, c), suggesting that the CMA-DYRK1A pathway may not intersect with the CMA-GLUL-Gln axis. Previous studies have demonstrated that GLS1 forms filamentous polymers under glutamine deficiency, which triggers cell apoptosis.^42^ In the present study, we extended these findings by demonstrating that administration of GLS1 inhibitors, including CB-839 and BPTES, significantly decreased L2A overexpression-induced apoptotic protein levels in senescent NPC (Fig. 6m). This suggested that GLS1 played a critical role in the apoptotic toxicity of senescent NPC following CMA activation. Furthermore, immunofluorescence staining revealed that GLS1 in senescent NPC formed filamentous aggregates upon L2A overexpression (Fig. 6n). Notably, treatment with CB-839 to inhibit GLS1 activity reduced these filamentous aggregates (Fig. 6n). In addition, TUNEL staining demonstrated that GLS1 inhibitors could rescue L2A overexpression-induced apoptosis in senescent NPC (Fig. S8a). Collectively, these findings indicate that CMA activation-mediated apoptosis in senescent NPC is associated with disruption of the GLUL-Gln axis and is partially attributable to the filamentous polymer toxicity of GLS1.

### CMA participates in the senolytic activity of senescent NPC by targeting GLUL degradation

**Fig. 7:**
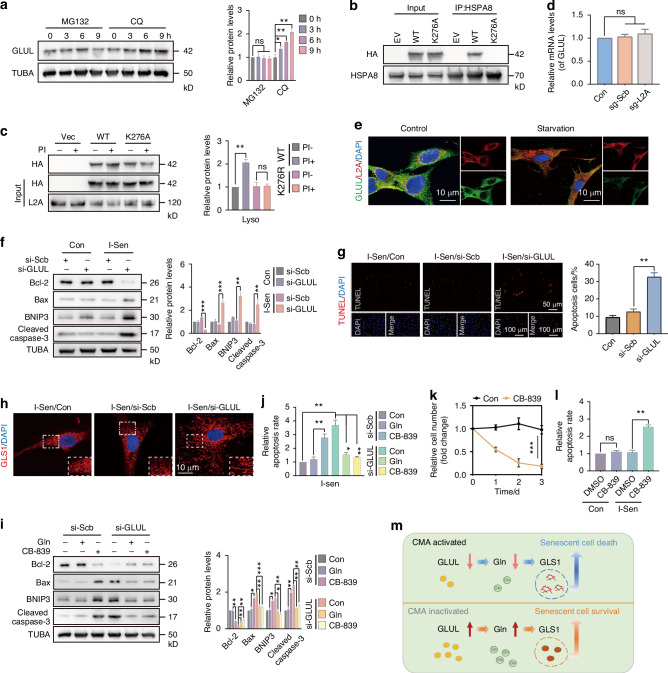
CMA participates in the senolytic activity of senescent NPC by targeting GLUL degradation. **a** The effect of MG132 or CQ on GLUL levels in NPC. **b** The protein level of HA-GLUL in the co-precipitate of HSPA8 in NPC transfected with WT- or K276A-GLUL plasmids. **c** Immunoblotting showing the effect of lysosomal enzyme inhibitor PI on HA-GLUL levels in lysosomes of NPC in WT or K276A groups. **d** RT-qPCR showing the mRNA levels of GLUL in NPC after L2A knockout. **e** Immunofluorescence showing the colocalization of GLUL and L2A in normal or starved NPC. **f** Western blot showing the levels of apoptosis-associated proteins in normal or I-Sen NPC after GLUL knockdown. **g** TUNEL staining showing the effect of GLUL knockdown on apoptosis of I-Sen NPC. **h** Immunofluorescence showing the effect of si-GLUL transfection on GLS1 filaments in I-Sen NPC. **i** The effect of Glutamine or CB-839 on the levels of apoptosis-related proteins in I-Sen NPC treated with or without GLUL knockdown. **j** Flow cytometry results showing the effect of Gln or CB-839 on I-Sen NPC apoptosis after GLUL knockdown. **k** Relative cell counts of I-Sen NPC treated with or without CB-839 after 1, 2 and 3 days. **l** The effect of CB-839 on apoptosis levels in normal or I-Sen NPC. **m** Schematic diagram showing that CMA activation promotes senolysis in senescent NPC via degradation of GLUL. All graphs show the mean ± SEM of at least three independent experiments. *P <0.05, **P <0.01, ***P <0.001, ns indicates not significant

We have demonstrated that GLUL overexpression can rescue CMA-induced apoptosis in senescent NPC. However, further evidence is required to establish GLUL as a CMA substrate. Through treatment with MG132 and CQ, we determined that GLUL degradation occurs via the lysosomal pathway, rather than the ubiquitin-proteasome system (Fig. 7a). Subsequently, we investigated whether altered CMA or macroautophagy activity influences GLUL protein levels. The result showed that activation of CMA by AR7 reduced GLUL levels, while CQ counteracted this effect of AR7 (Fig. S8b). In a time-dependent study using 3MA to block macroautophagy, SQSTM1 levels increased progressively, while GLUL levels remained unchanged, and even decreased at 9 h due to crosstalk between CMA and macroautophagy (Fig. S8c). Knockdown of L2A, but not ATG7, significantly decreased GLUL levels in NPC lysosomes (Fig. S8d). Sequence analysis identified KLSKR at position 276 as a potential CMA recognition motif in GLUL (Fig. S8e). To validate this, we generated HA-GLUL with a K276A mutation. Immunoprecipitation assays revealed that HA-GLUL co-immunoprecipitated with HSPA8 in the WT group, but not in the K276A mutant group (Fig. 7b), indicating that the 276-KLSKR motif is essential for HSPA8-mediated recognition of GLUL. Furthermore, in NPC transfected with WT or K276A plasmids, following treatment with PI (a lysosomal enzyme inhibitor), HA-GLUL levels were increased in the WT group, but remained unchanged in the K276A group (Fig. 7c). Notably, while L2A knockout increased GLUL protein levels, it did not affect GLUL transcription (Fig. 7d). Additionally, molecular fluorescence imaging demonstrated colocalization of GLUL and L2A was enhanced under starvation (Fig. 7e). Collectively, these findings establish GLUL as a specific substrate of CMA.

Further, we investigated the role of GLUL in NPC apoptosis. In normal NPC, GLUL knockdown did not induce apoptosis. However, in senescent NPC, GLUL knockdown significantly induced apoptosis, as shown by increased Bax/Bcl-2 ratio, BNIP3 expression, and cleaved caspase 3 levels (Fig. 7f). TUNEL staining further demonstrated that si-GLUL transfection effectively promoted apoptosis in senescent NPC (Fig. 7g), consistent with the role of CMA activation in promoting apoptosis of senescent NPC. Notably, we observed that GLS1 morphology transitioned to filamentous structures following GLUL knockdown (Fig. 7h), which could be attributed to disrupted glutamine synthesis.^42^ Our findings revealed that these GLS1 filaments contributed to apoptotic toxicity in senescent NPC. GLUL knockdown-induced apoptosis in senescent NPC could be rescued by treatment with the GLS1 inhibitor CB-839 or glutamine (Fig. 7i, j). Interestingly, while CB-839 could rescue CMA or si-GLUL-induced apoptosis of senescent NPC, the application of GLS1 inhibitors alone in senescent NPC also promoted apoptosis (Fig. 7k, l). This phenomenon may be associated with the blockade of glutamine catabolism. In contrast, GLS1 inhibitors did not induce apoptotic toxicity in normal NPC (Fig. 7k, l). Collectively, these results demonstrate that CMA regulates the glutamine-addicted survival of senescent NPC by promoting GLUL degradation (Fig. 7m). The adaptive reprogramming of the GLUL/Gln/GLS1 axis by CMA in senescent NPC provides new insights for developing senolytic strategies.

## Discussion

CMA is associated with cellular senescence and degenerative diseases,^43^ but its role and mechanism in SASP-mediated senescence self-amplification feedback need further study. IL1B is the core inflammatory factor of SASP. We found that IL1B treatment can directly induce CMA inhibition, and activation of CMA can counteract the senescence and degeneration of intervertebral discs caused by IL1B; on the other hand, the knockdown of CMA directly induces the phenotype of IDD, which has been verified by imaging and histology. Through TMT-MS after L2A knockout, we inferred that the significantly increased DYRK1A may affect the premature senescence process of NPC. Structural and functional analyses confirmed that DYRK1A is the true substrate of CMA, rather than macroautophagy or the UPS system. DYRK1A is a key gene on chromosome 21 that causes the premature aging phenotype of Down syndrome. It is widely expressed in organs and tissues such as the brain, heart, lungs, and skeletal muscle.^44^ DYRK1A can phosphorylate a variety of substrates, including transcription factors, cell cycle proteins, and histone acetyltransferases, thereby regulating cell proliferation, differentiation, apoptosis, and senescence.^45^ Overexpression of DYRK1A has been shown to be associated with the occurrence of a variety of degenerative diseases, including Alzheimer's disease and Parkinson’s disease.^46,47^ Many studies have found that activation of DYRK1A promotes cell cycle arrest,^48^ which may involve the functional preference of its phosphorylated substrates. DYRK1A can promote mammalian cell cycle quiescence and senescence through the assembly of the DREAM complex.^49^ Here, we found that the increase of DYRK1A drives the premature senescence process of NPC under CMA inhibition, including cell cycle arrest and expression of senescence-related proteins. By combining RNA-seq of DYRK1A overexpression and IP-MS of DYRK1A-binding proteins for TF analysis, we identified FOXC1 as the only target protein that intersects these two gene sets and the GO gene set that promotes cell cycle arrest. FOXC1 is a forkhead-box transcription factor that can regulate the expression of multiple genes and participate in cell development, differentiation, and senescence. DNA microarray data showed that FOXC1 is a key transcription factor associated with senescence in mesenchymal stem cells.^50^ We found that FOXC1 itself is rich in a large number of serine/threonine and tyrosine phosphorylation sites, and verified FOXC1 as a phosphorylation target of DYRK1A. Our data indicate that phosphorylation of FOXC1 by DYRK1A promotes its nuclear translocation. Functionally, the nuclear translocation of phosphorylated FOXC1 is necessary for DYRK1A to promote cell cycle arrest in NPC, which affects the expression of a series of cell cycle regulatory genes, including MKI67, PCNA and P27, as well as senescence-related proteins P53, P21 and P16. Therefore, we confirmed that CMA inhibition can promote premature senescence of NPC under the senescent microenvironment through the DYRK1A/FOXC1 pathway.

As a key target for regulating cell growth and development, DYRK1A is considered to be an important drug target in multiple therapeutic areas such as neurodegenerative diseases, pancreatic and cardiac regeneration, and the development of inhibitors targeting DYRK1A is becoming a hot topic in current research.^51^ Many small-molecule DYRK1A inhibitors have been developed, such as natural inhibitors, such as Harmine, EGCG, and leucettines, and synthetic inhibitors, such as quinazoline and benzothiazole.^45^ Coincidentally, some natural DYRK1A inhibitors have previously been used as potential treatments for IDD. EGCG is a tea polyphenol substance with a high content in green tea, which can bind to DYRK1A kinase in a non-competitive form in vitro. EGCG has been shown to exhibit significant cytoprotective effects on NPC in ROS and inflammatory environments,^52^ and to a certain extent improve acupuncture-induced rat intervertebral disc degeneration, as well as maintain intervertebral disc height and NP tissue structure.^53^ In addition, DYRK1A may also induce the NPC senescent process through other mechanisms. NF-κB signaling is the main signaling pathway that stimulates NPC senescence and the emergence of SASP.^10,32^ Studies have found that DYRK1A can phosphorylate TRAF3 and interfere with its function of mediating NIK degradation, thereby promoting the activation of the NF-κB pathway.^54^ Inhibition of DYRK1A using Harmine or siRNA significantly reduced the production of proinflammatory factors in LPS-stimulated neurons, which was related to the inhibition of the TLR4/NF-κB signaling pathway.^55^ We validated that the CMA-DYRK1A-FOXC1 axis also intersects with the NF-κB signaling pathway in NPC, suggesting that DYRK1A may control a discrete senescence regulatory program. In fact, using DYRK1A as a target for regulating the cell cycle has become a new hot topic, especially DYRK1A inhibitors have become the direction of future drug development in promoting cardiomyocyte and β-cell regeneration. High-throughput chemical screening revealed that Harmine-mediated inhibition of DYRK1A can induce human pancreatic β-cell replication in vitro and in vivo, increase islet mass, and improve glycemic control.^51,56^ DYRK1A inhibition can also activate cell cycle-associated proteins and CDKs, accompanied by a decrease in key cell cycle inhibitors (CDKN1C and CDKN1A), thereby promoting normal human β-cell replication.^57^ DYRK1A knockdown also leads to robust activation of the cardiomyocyte cell cycle and promotes enhanced expression of many genes that control cell proliferation.^48^ Both conditional deletion and pharmacological inhibition of DYRK1A can induce cardiomyocyte cell cycle activation and cardiac repair in mice, thereby improving cardiac function. Christian et al. reported that DYRK1A is a novel negative regulator of D-cyclin-mediated Rb/E2f signaling, and its overexpression leads to impaired cardiomyocyte cell cycle progression.^58^ In summary, these studies support that DYRK1A inhibition can alleviate inflammation-induced cell cycle arrest, which is consistent with our findings in the context of IL1B-induced NPC premature senescence. Although DYRK1A inhibitors are under development, few studies have investigated the mechanism of endogenous inhibition of DYRK1A activity. Here, we found that CMA activation can inhibit DYRK1A activity by targeting DYRK1A for degradation, indicating that CMA activation can serve as an endogenous inhibitory mechanism of DYRK1A. In the future, this will help promote the development of CMA activators as potential DYRK1A inhibitors, although they themselves have some similar functional applications in some disease treatment areas, including neurodegeneration and inflammation.

We performed total RNA sequencing on senescent NPC under the action of CMA activator, and the results showed that CMA activation significantly promoted the apoptotic pathway. We verified that CMA activation can promote apoptosis in senescent NPC, accompanied by the reduction of senescence-related indicators. Our previous mass spectrometry analysis and previous studies by others have pointed to the significant effects of CMA on cellular metabolism,^20,59^ including glucose, lipid,^18^ and fatty acid metabolism^19^. Here, we found evidence that CMA can regulate glutamine metabolism. Glutamine synthetase GLUL was significantly increased in NPC after L2A knockout. Normally, the survival of senescent cells depends on glutamine flux, and its inhibition has been shown to be a promising strategy to induce senescent cell death and improve multiple age-related diseases.^9^ In senescent NPC, we found that reactivation of CMA induced glutamine deficiency. Glutamine addiction is a characteristic of senescent cells for survival, and its imbalance may explain how CMA promotes the apoptotic susceptibility of senescent NPC, but not normal NPC. Previous studies have reported that glutamine supplementation is associated with reduced disc aging and degeneration.^60^ We found that senescent NPC have a high demand for glutamine flux and exhibit high expression of the GLUL/Gln/GLS1 axis. CMA activation leads to inhibition of glutamine synthesis in senescent cells, which increases the susceptibility of senescent NPC to apoptosis. In addition, we found that CMA activation-induced apoptosis of senescent NPC is also associated with cytotoxicity mediated by GLS1 filament aggregation. The ability of GLS1 to form filaments is directly related to its maximum catalytic activity.^61,62^ Previous studies have found that GLS1 filaments are required for glutamine deprivation-induced apoptosis,^42^ which is related to asparagine deficiency promoting mitochondrial Bax translocation. In summary, the difference in glutamine metabolism between normal cells and senescent NPC may explain why CMA activation plays a protective role in normal cells from apoptosis,^63^ but conversely, exerts a pro-apoptotic effect in aged cells. In fact, CMA also plays a double-edged sword role in ferroptosis. Under certain conditions, the activation of CMA is involved in the execution of ferroptosis, which is related to the degradation of GPX4.^37^ In other cases, CMA prevents the production of lethal lipid substances and the occurrence of ferroptosis by degrading ACSL4.^38^ Therefore, the effect of CMA on apoptosis may also involve differences in cells and conditions. In the future, it is also necessary to explore the effects and molecular mechanisms of CMA on the survival of senescent cells in more cell types and conditions.

**Fig. 8:**
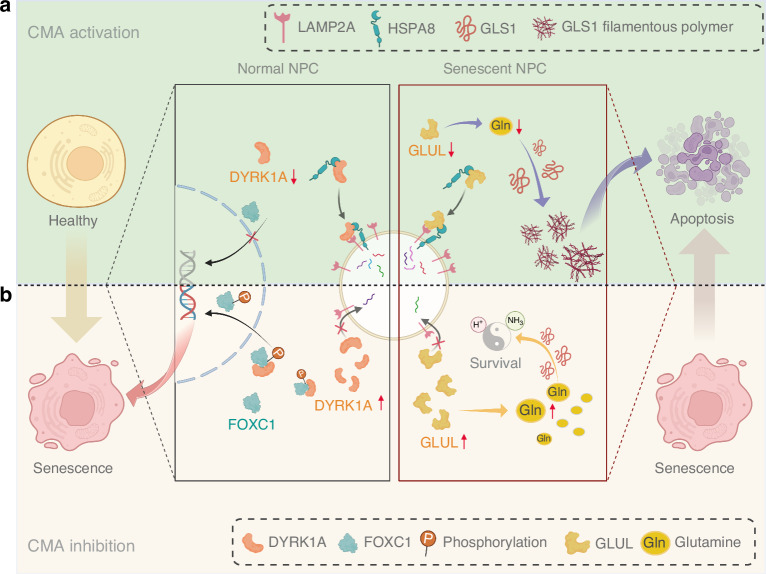
Schematic representation of the dual mechanisms directed by CMA to balance the premature senescence of normal NPC and the clearance of senescent NPC. CMA is essential for maintaining the youthfulness of the intervertebral disc. It acts as a biological rheostat to dynamically regulate the fate of normal and senescent NPC in the intervertebral disc: **a** In normal NPC, CMA inhibition promotes premature senescence through the DYRK1A-FOXC1 axis; in senescent NPC, CMA inhibition participates in the remodeling of glutamine metabolism and serves as one of its pro-survival mechanisms; **b** CMA activation alleviates the NPC senescence induced by elevated DYRK1A, while reducing the glutamine level in senescent NPC through GLUL degradation, thereby increasing their sensitivity to apoptosis

Normally, senescent cells are cleared within a few days under the surveillance of immune cells.^64^ However, due to the natural lack of blood supply in the intervertebral disc, senescent NPC easily exceed the threshold burden and continue to accumulate. The accumulation and persistence of senescent cells can induce a chronic, low-grade, profibrotic, sterile inflammatory state,^65^ causing dysfunction of adjacent and distant non-senescent cells and further amplifying the accumulation of senescent cells in the feedforward loop, leading to impaired tissue function and reduced regenerative capacity.^66,67^ In this context, the senolytic strategy of targeted clearance of senescent cells has been shown to widely improve the local microenvironment and organ health of the intervertebral disc. For example, long-term treatment with the anti-aging drugs dasatinib and quercetin can improve age-dependent IDD in mice.^34^ Local administration of senolytic drugs can also reduce the expression of proinflammatory cytokines and matrix proteases in the disc, thereby inhibiting the IDD process in rats.^35^ Although conventional senolytic therapy has shown potential in delaying the senescence process and treating age-related diseases, it also has some challenges, such as off-target cytotoxicity to normal tissues. Long-term systemic use of senolytic drugs may cause side effects such as anemia, thrombocytopenia, and neutropenia.^64^ Here, we observed huge differences in the role of CMA activation on the survival and function between normal and senescent NPC, which may benefit the overall anti-aging and anti-degeneration process of the intervertebral disc. CMA activation not only helps normal NPC fight against inflammation-induced senescence, but it also induces the apoptosis process of senescent NPC, thereby attenuating the continued harm of SASP. This contrasting effect distinguishing the characteristics of normal and senescent cells is expected to make CMA activators a potentially safer complementary senolytic therapy that can delay the aging process of normal NPC while helping to target and eliminate already senescent NPC, thereby maximizing the improvement of the senescent microenvironment of the intervertebral disc and maintaining its youthful state (Fig. 8).

At present, the treatment methods for IDD mainly include conservative treatment (such as physical therapy, drug therapy) and surgical treatment (such as discectomy, intervertebral fusion). Conservative treatment is usually used to relieve symptoms, but it cannot act on the process of IDD. Although surgical treatment can effectively relieve pain, it has problems such as large trauma and many postoperative complications. In recent years, targeted therapy based on cell and molecular mechanisms has gradually become a research hotspot, such as stem cell therapy and gene therapy.^68^ However, these methods still face many challenges in clinical application, such as low survival rate of stem cells and safety issues of gene therapy. In contrast, CMA activators have been shown to improve aging-related degenerative diseases in various models, such as neurodegenerative diseases,^69^ cardiovascular diseases,^20,70^ liver metabolic abnormalities^71^ and other disease models.^72,73^ Our study has shown that CMA can directly intervene in the senescence-SASP cycle network of intervertebral disc cells by regulating transcriptional programs and metabolic pathways, and can selectively remove senescent NPC, which has potential advantages in etiological treatment of IDD. In addition, CMA activators can protect normal cells from damage caused by inflammation and oxidative stress. For example, CMA can degrade the components of inflammasomes to inhibit the release of IL1B,^74,75^ and CMA can promote the degradation of keap1 to enhance the antioxidant signal of Nrf2.^22^ However, these mechanisms need to be further verified in NPC. Although CMA activators have shown great potential in the treatment of IDD, their clinical application still faces some challenges. First, the pharmacokinetic and pharmacodynamic properties of CMA activators need further study to ensure their stability and effectiveness in vivo. Secondly, the mechanism of action of CMA activators involves multiple cellular signaling pathways. Although CMA is defined as a cell protection mechanism in most studies,^18^ CMA is still pointed out to be related to ferroptosis induction in a few studies.^37^ Therefore, its long-term use safety and potential off-target effects still need to be evaluated. In addition, the special anatomical structure of the intervertebral disc (such as a lack of blood supply) may affect the local delivery and distribution of drugs, which requires the development of more efficient drug delivery systems to overcome this. From the perspective of clinical application, the development of CMA activators requires multidisciplinary collaboration, including medicinal chemistry, pharmacology, and clinical medicine, and further preclinical and clinical trials are needed to verify their efficacy and safety. In addition, the combination of nanotechnology and targeted delivery systems is expected to increase the local concentration and bioavailability of CMA activators in the intervertebral disc, thereby enhancing their therapeutic effects. In the future, combined treatment strategies based on CMA activators (such as combined with stem cell therapy or anti-inflammatory drugs) may also become an important direction for the treatment of IDD.

## Materials and methods

### Specimen acquisition, ethics, and cell isolation

We collected 50 degenerated disc samples from patients undergoing spinal surgery (age range 25–73 years, including 25 women and 25 men). The degree of degeneration of these samples was assessed according to the Pfirrmann grade. For comparison, we also obtained 48 samples from patients undergoing surgery for thoracolumbar spine injury or scoliosis (age range 18–37 years, including 25 women and 23 men). This study followed the guidelines of the Declaration of Helsinki, and written consent was obtained from all participants under the advice of the committee. In addition, the protocol of this study was formally approved by the Ethics Committee of Tongji Medical College, Huazhong University of Science and Technology.

For the extraction of primary cells, healthy human disc nucleus pulposus (NP) tissue was first cut into 1 mm^3^ pieces, and then digested with 0.2% collagenase type II (product number 2275MG100, BioFroxx, Germany) at 37 °C for about 8 h to completely separate the cells from the tissue. The digested samples were centrifuged at 1 000 r/min for 5 min and then cultured in Dulbecco’s modified Eagle’s medium/nutrient mixture F-12 (Gibco, USA) containing 10% fetal bovine serum (Cat. No. 10099-141, Gibco, Invitrogen, New York, USA) and 1% penicillin-streptomycin (Cat. No. V900929, Sigma, USA) at 37 °C and 5% CO_2_. When the cells grew to 75% confluence, we digested them with 0.25% trypsin (Cat. No. 25200114, Gibco, Invitrogen, USA) and subcultured them. We used the second generation of NP cells (NPC) for subsequent experiments.

### Drugs and cell processing

AR7 (#HY-101106), Chloroquine/CQ (#HY-17589A), 3MA (#HY-19312), BPTES (#HY-12683), apoptosis inducer-10/AI-10 (#HY-146255), IL1B (#HY-P73149), All-trans Retinoic acid/ATRA (#HY-14649), Rapamycin/Rap (#HY-10219) and MG132 (#HY-13259) were purchased from MedChemExpress company. CB-839 (#S7655), QX77 (#S6797), Harmine/Har (#S3868) and nutlin-3a (#S8059) were purchased from Selleck company. In terms of I-Sen NPC, we treated NPC with 10 ng/mL IL1B (#IL038, Sigma, USA) for 48 h to induce cellular senescence. As another positive control, we also cultured cells with nutlin-3a (10 μmol/L, 48 h) to induce senescence (N-Sen). NPCs at passage nine were used as a model of replicative senescence (R-Sen).

### Senescence-associated-(SA-)β-galactosidase staining

We used Fluorescein diβ-D-galactopyranoside (FDG) (#HY-101895, MedChemExpress, USA) to detect SA-β-gal activity. We evenly distributed the reaction buffer to each well and then added 2 mmol/L FDG. The cell plate was incubated at 37 °C for 24 h in the dark without adding carbon dioxide to the incubator. After one day of incubation, a fluorescence microscope (Olympus, Japan) was used to observe and record the fluorescence images of the cells. In addition, we used an SA-β-gal-based near-infrared Senescence-Tracker (# C0603, Beyotime, Shanghai) to track senescent cells in vivo or in vitro. For in vivo senescence imaging in rats, 10 μL of 10 μmol/L Senescence-Tracker was injected into the coccygeal intervertebral disc, and the abundance of senescent cells was detected by infrared imaging two weeks later. For in vitro experiments, NPC were incubated with cell culture medium containing Senescence-Tracker (10 μmol/L) at 37 °C for 24 h, and then live cell imaging was performed under a confocal microscope. ImageJ was used to quantify the average cell fluorescence intensity. To ensure the reliability of the experimental results, we performed at least three replicates.

### Western blot and Immunoprecipitation

First, we placed the samples in RIPA lysis buffer (#BL504A, Biosharp, China) containing protease and phosphatase inhibitor cocktail (#P1049, Beyotime, China) and PMSF (#ST506, Beyotime, China) at 4 °C for lysis. The ultrasonically treated samples were centrifuged to separate and collect the clear supernatant. Then, the protein content in the supernatant was determined using the BCA protein quantification kit (#23225, Thermo Scientific, USA). The protein samples were mixed with loading buffer (#LT101, Epizyme, China) and heated to boiling for 10 min to achieve complete protein denaturation. After that, the proteins were electrophoresed by SDS-polyacrylamide gel electrophoresis and transferred to a PVDF membrane. For immunoprecipitation, cells were lysed using IP-specific cell lysis buffer (#P0013, Beyotime, China) containing protease and phosphatase inhibitors (#P1049, Beyotime, China) and PMSF (#ST506, Beyotime, China). Subsequently, immunoprecipitation was performed using magnetic beads with anti-protein A/G (#HY-K0202, MedChemExpress, USA) and specific antibodies. The separated target proteins were transferred to the membrane by SDS/PAGE electrophoresis. After incubation in blocking buffer for 1 h, the membrane was incubated with specific primary antibodies (see the attached Table 1) at 4 °C overnight. Subsequently, the membrane was washed with TBST (Tris-buffered saline containing 0.1% Tween 20) for 30 min and incubated with HRP-labeled secondary antibodies for 1 h at room temperature. Finally, after three washes, the proteins were visualized using an enhanced chemiluminescence reagent (#HY-K1005, MedChemExpress, USA) and the ChemiDoc MP system (Bio-Rad, USA), and the abundance of protein bands was quantified using ImageJ software.

**Table 1:** Primary antibodies

Product name	Catalog	Manufacturer	Dilution Ratio
LAMP2A	ab125068	Abcam	1:1 000 (WB); 1:200 (IF)
DYRK1A	ab65220	Abcam	1:1 000 (WB); 1:100 (IF)
P53/TP53	10442-1-AP	Proteintech	1:5 000 (WB); 1:200 (IF)
P21/CDKN1A	10355-1-AP	Proteintech	1:2 000 (WB); 1:200 (IF)
P16/CDKN2A	ab270058	Abcam	1:2 000 (WB); 1:100 (IF); 1:500 (FC)
ATG7	10088-2-AP	Proteintech	1:2 000 (WB)
IL1B	16806-1-AP	Proteintech	1:2 000 (WB)
SQSTM1	18420-1-AP	Proteintech	1:2 000 (WB)
TUBA	M20005	Abmart	1:20 000 (WB)
FOXC1	55365-1-AP	Proteintech	1:1 000 (WB); 1:200 (IF)
β-actin	81115-1-RR	Proteintech	1:5 000 (WB)
LAMP1	21997-1-AP	Proteintech	1:2 000 (WB); 1:200 (IF)
HA	51064-2-AP	Proteintech	1:3 000 (WB)
P-Ser/Thr	ab17464	Abcam	1:1 000 (WB)
P-Tyr	P1869	Sigma-Aldrich	1:1 000 (WB)
Bcl-2	381702	Zenbio	1:1 000 (WB)
Bax	50599-2-Ig	Proteintech	1:2 000 (WB)
BNIP3	68091-1-Ig	Proteintech	1:2 000 (WB)
Cleaved caspase 3	25128-1-AP	Proteintech	1:2 000 (WB)
IL-6	500286	Zenbio	1:1 000 (WB)
IL-8	R30084	Zenbio	1:1 000 (WB)
TNF-α	60291-1-Ig	Proteintech	1:2 000 (WB)
MMP3	17873-1-AP	Proteintech	1:1 000 (WB)
GLUL	66323-2-Ig	Proteintech	1:2 000 (WB); 1:200 (IF)
GLS1	12855-1-AP	Proteintech	1:2 000 (WB); 1:200 (IF)
Galectin-3	82024-1-RR	Proteintech	1:200 (IF)
RelA/NF-κB p65	10745-1-AP	Proteintech	1:2 000 (WB); 1:200 (IF)
IKBA	10268-1-AP	Proteintech	1:2 000 (WB); 1:200 (IF)

### Real-time quantitative reverse transcription PCR (RT-qPCR)

We used an RNA extraction kit (#RC101, Vazyme, China) to isolate RNA from cultured cells or NP tissues. According to the manufacturer's instructions, we used a cDNA kit (#AE311-02, TransScript, China) to reverse transcribe RNA, and then used qPCR super mix reagent (#AQ601-01, TransScript, China) to perform RNA fluorescence amplification and RT-qPCR analysis. The primer sequences required for RT-qPCR are detailed in the attached Table 2.

**Table 2:** Primer sequences

Gene	Primers
Homo P53	Forward 5′-CAGCACATGACGGAGGTTGT-3′
	Reverse 5′-TCATCCAAATACTCCACACGC-3′
Homo P21	Forward 5′-TGTCCGTCAGAACCCATGC-3′
	Reverse 5′-AAAGTCGAAGTTCCATCGCTC-3′
Homo P16	Forward 5′-GATCCAGGTGGGTAGAAGGTC-3′
	Reverse 5′-CCCCTGCAAACTTCGTCCT-3′
Homo IL1B	Forward 5′-AGCTACGAATCTCCGACCAC-3′
	Reverse 5′-CGTTATCCCATGTGTCGAAGAA-3′
Homo DYRK1A	Forward 5′-CAAACACGACACTGAAATGAAGT-3′
	Reverse 5′-GGAAGTTGGTGTTTCTCAACAA-3′
Homo IL-6	Forward 5′-ACTCACCTCTTCAGAACGAATTG-3′
	Reverse 5′-CCATCTTTGGAAGGTTCAGGTTG-3′
Homo IL-8	Forward 5′-AGACAGCAGAGCACACAAGC-3′
	Reverse 5′-ATGGTTCCTTCCGGTGGT-3′
Homo-LAMP2A	Forward 5′-TGTCTGGAGCATTTCAGATAAA-3′
	Reverse 5′-ATGGGCACAAGGAAGTTG-3′
Homo LAMP2B	Forward 5′-AGAGTGTTCGCTGGATGATG-3′
	Reverse 5′-TGCCAATTACGTAAGCAATCA-3′
Homo LAMP2C	Forward 5′-AAGGGTTCAGCCTTTCAATG-3′
	Reverse 5′-ACAATTATAAGGAAGCCCAAGG-3′
Homo GLUL	Forward 5′-GTGTGTGGAAGAGTTGCCTG-3′
	Reverse 5′-CTGCAGGCCTTCGATTGTAC-3′
Homo GAPDH	Forward 5′-ACAACTTTGGTATCGTGGAAGG-3′
	Reverse 5′-GCCATCACGCCACAGTTTC-3′
Homo MKI67	Forward 5′-ACGCCTGGTTACTATCAAAAGG-3′
	Reverse 5′-CAGACCCATTTACTTGTGTTGGA-3′
Homo PCNA	Forward 5′-TCTGAGGGCTTCGACACCTA-3′
	Reverse 5′-TCATTGCCGGCGCATTTTAG-3′
Homo p27	Forward 5′-GGCTCCGGCTAACTCTGA-3′
	Reverse 5′-TCTTCTGTTCTGTTGGCTCTTT-3′

### Immunofluorescence staining and TUNEL staining

The cells or tissue samples were fixed with 4% formaldehyde solution for 30 min, and 0.5% Triton X-100 was applied for 10 min to permeabilize the cell membrane. After each fixation or permeabilization, the samples were washed with PBS three times, each for 5 min. Then, they were incubated with 2% BSA solution for half an hour at room temperature to block nonspecific binding sites. Samples were incubated with the following primary antibodies overnight at 4 °C or for 2 h at room temperature: Anti-LAMP2A (1:200, ab125068, Abcam), Anti-P53 (1:200, 10442-1-AP, proteintech), Anti-P21 (1:200, 10355-1-AP, proteintech), Anti-P16-INK4A (1:100, ab270058, Abcam), Anti-DYRK1A (1:100, ab65220, Abcam), Anti-FOXC1 (1:200, 55365-1-AP, proteintech), Anti-Galectin-3 (1:200, 60207-1-Ig, proteintech), Anti-LAMP1 (1:200, 67300-1-Ig, proteintech), Anti-GLS1 (1:200, R24458, Zenbio), Anti-GLUL (1:200, 66323-1-Ig, proteintech). After three PBS washes, the samples were incubated with fluorescently labeled secondary antibodies for 1 h at room temperature. For TUNEL staining, a kit (#C1089, Beyotime, China) was used. Specifically, after fixing the cells with 4% paraformaldehyde for 30 min, they were washed once with PBS and then permeabilized with PBS containing 0.3% Triton X-100 and incubated at room temperature for 5 min. Subsequently, 50 μL of TUNEL detection solution was added to the samples and incubated at 37 °C in the dark for 60 min. Before observation, all slides were stained for cell nuclei with DAPI (#C1002, Beyotime, China) and sealed with an anti-fluorescence quencher. Finally, imaging analysis was performed using a fluorescence microscope or confocal microscope (Olympus, Japan).

### Animal model of disc injection and evaluation of the extent of IDD

We performed surgery on Sprague-Dawley rats (weight 250 ± 20 g) following the previous method. Specifically, the rats were housed under SPF conditions with an ambient temperature of 24 °C and a humidity of approximately 50%. First, we anesthetized the rats with 3% pentobarbital (w/v, 2 mL/kg). During the surgery, the Co7/8 discs of the rats were vertically injected with a 33-G needle without the knowledge of the surgeon. The drugs injected included IL1B, PBS, DMSO, AR7, and Lentivirus containing EV, Lamp2a, sh-Scb, and sh-Lamp2a. Each injection volume was 2 μL, and the depth was approximately 5 mm. The injection frequency was once a week for one month and then maintained for one month. All animals were allowed to move freely without weight-bearing restrictions. In the eighth week of surgery, we performed magnetic resonance imaging (MRI) on all rats for imaging and analysis. During the scanning process, the anesthetized rats were kept in a prone position with their tails held straight. We determined the degree of disc degeneration and the disc height index (DHI) according to the Pfirrmann score. Subsequently, the rats were anesthetized with an overdose of 3% pentobarbital, and the specimens were collected. After fixation, decalcification, dehydration, and paraffin embedding, the specimens were cut into 4-μm-thick sections and subjected to H&E staining, Safranin O-Fast Green staining, and Masson staining. These tissue-stained sections were used for histological scoring of the degree of disc degeneration in rats, and the evaluation method was as described previously.^76^ The animal experiments were conducted in accordance with the “3 R” principle and were approved by the Animal Ethics Committee with approval number HLK-20240315-001.

### Cell cycle and proliferation capacity detection

We used the cell cycle and apoptosis detection kit (#C1052, Beyotime, China) for cell cycle analysis. Following the manufacturer's instructions, cells were collected and suspended in 70% ethanol and fixed at 4 °C for 12 h. Subsequently, cells were washed with pre-cooled PBS and stained with propidium iodide (PI). Red fluorescence signals were detected by flow cytometry at a wavelength of 488 nm, and light scattering analysis was performed. FlowJo V10 software (BD Biosciences, Franklin Lakes, New Jersey) was used to analyze cell cycle data, especially the proportion of cells in S phase. The detection of cell proliferation marker BrdU was done by BrdU cell proliferation detection kit (#ab287841, Abcam) according to the manufacturer’s instructions. In a 96-well plate, 5 000 untreated or transfected NPC were placed in each well and cultured for 48 h after treatment. BrdU solution was added to the designated wells and incubated at 37 °C for 4 h. After removing the culture medium, 100 μL of fixative solution was added to each well and incubated at room temperature for 30 min. Then, add 100 μL of BrdU detection solution to each well and incubate for 1 h with gentle shaking. After washing, add 100 μL of antibody solution to each well and incubate for 1 h at room temperature. Add 100 μL of TMB substrate to each well and measure the absorbance at 650 nm after 30 min. To stop color development, add 100 μL of stop solution to each well and measure the absorbance at 450 nm.

### Tandem mass tag (TMT) based mass spectrometry (TMT-MS)

After NPC were transfected with lentiviral vectors of sg-LAMP2A or sg-Scb, cells were collected by centrifugation and lysed with SDT buffer (containing 4% SDS, 100 mmol/L Tris-HCl, 1 mmol/L DTT, pH 7.6) to extract proteins. The protein content was determined using the BCA protein assay kit (#5000001, Bio-Rad, USA). We labeled 100 μg of peptides from each sample with TMT labeling reagent according to the instructions. Mass spectrometry analysis was performed on a Q Exactive mass spectrometer (#IQLAAEGAAPFALGMAZR, Thermo Scientific, USA) connected to Easy-nLC (Proxeon Biosystems) with an analysis time of 60/90 min. We identified proteins by searching the spectral data in the Uniprot database and analyzed the relative expression levels of proteins after LAMP2A knockout.

### RNA sequencing (RNA-seq)

We used TRIzol reagent (#T9424, Sigma-Aldrich, USA) to extract total RNA from tissue cells. Subsequently, we used Nanodrop ND-2000 (#ND-2000, Thermo Scientific, USA) and Agilent Bioanalyzer 4150 (#G2992AA, Agilent Technologies, California, USA) to detect the quality of RNA samples by A260/A280 absorbance ratio and RIN value. The construction of the PE library followed the instructions of the ABclonal mRNA-seq Lib Prep Kit (#RK20350, ABclonal, China). We used oligo (dT) magnetic beads to purify mRNA from 1 μg of total RNA, and then performed mRNA fragmentation in ABclonal First Strand Synthesis Reaction Buffer. Next, the first strand of cDNA was synthesized using random primers and reverse transcriptase (RNase H) with the mRNA fragment as a template, and then the second strand of cDNA was synthesized using DNA polymerase I, RNAse H, buffer, and dNTPs. We connected the synthesized double-stranded cDNA fragments to the adapter sequence for PCR amplification. After the amplification product was purified, we used an Agilent Bioanalyzer 4150 to assess the quality of the library. Finally, we used the NovaSeq 6000 (or MGISEQ-T7) sequencing platform, PE150 read length for sequencing. The data generated from the Illumina platform will be used for bioinformatics analysis.

### Mass spectrometry-based immunoprecipitation (IP-MS) proteomics analysis

To identify interacting proteins of DYRK1A, we extracted cell lysates from NPC overexpressing DYRK1A and performed co-immunoprecipitation experiments using protein A/G magnetic beads conjugated with specific antibodies, and then collected the beads. The beads were washed three times with pre-cooled PBS solution and then incubated at 95 °C for 10 min in a reaction buffer containing 1% SDC, 100 mmol/L Tris-HCl (pH 8.5), 10 mmol/L TCEP, and 40 mmol/L CAA to complete protein denaturation, cysteine reduction, and alkylation. The eluted samples were trypsinized overnight at 37 °C. After that, we used self-prepared SDB desalting columns to purify the peptides. We acquired mass spectrometry data on a Q Exactive HF-X mass spectrometer and Easy-nLC 1200 system according to the instructions. The raw mass spectrometry data were analyzed by the Andromeda database search algorithm of MaxQuant software (version 1.6.6). We used the “proteingroups.txt” file output by MaxQuant for further data processing. The mean and standard deviation (SD) of protein expression changes were calculated, and the mean ± 1.64 times SD was used as the judgment threshold. Proteins exceeding this threshold were identified as proteins with significant differences between groups.

### RNA interference and plasmid transfection

Wuhan Miaoling designed and provided plasmids expressing wild-type and mutant human DYRK1A. Lenti-Easy Packaging Mix provided by GENECHEM (Shanghai, China) was used to package and condense wild-type and mutant DYRK1A plasmids. After confirming the effective transfection of NPC, we performed the subsequent experimental steps. Knockdown of LAMP2A, ATG7, GLUL, and FOXC1 in NPC was achieved by siRNA, and siRNA and its control siRNA (si-Scb) were provided by RIBOBIO (Guangzhou, China) and transfected using riboFECT™ CP (#C10511, RIBOBIO, China). Cas9-dependent knockdown of human LAMP2A was achieved by lentiviral-packaged sgRNA. Knockdown of rat-Lamp2a was achieved by lentiviral-packaged shRNA. Plasmids for overexpression of rat-Lamp2a were also constructed and lentivirally packaged by GENECHEM. The sequences of siRNA and sgRNA are listed in detail in Tables 3, 4.

**Table 3:** SiRNA sequences

Sequence name	target-sequence (sense of 5′-3′)
homo-si-LAMP2A	CTGGGATGTTCTTGTACAA
homo-si-ATG7	GAACGAGTATCGGCTGGAT
homo-si-FOXC1	CGGACAAGAAGATCACCCT
homo-sh-FOXC1	GGGAAACTGTATTAATCTTAT
rat-sh-FOXC1	GAATGGGAATAGTAGCTGTCA
homo-si-GLUL	GCACGUGUCUUCUCAAUGATT
homo-si-BNIP3	GGAATTAAGTCTCCGATTA
rat-sh-BNIP3	CCATCTCTGTTACTGTCTCAT
rat-sh-Lamp2a	CTGCAATCTGATTGATTA

**Table 4:** Sg-RNA sequences

Sequence name	target-sequence
homo-LAMP2A sgRNA -F	5′-CACCGGTGTTGCTGGCTTATTTTAT-3′
homo-LAMP2A sgRNA -R	5′-AAACATAAAATAAGCCAGCAACACC-3′

### CMA reporter plasmids and assay of CMA activity

Based on previous studies,^24^ we used the photoconvertible reporter plasmid pSIN-PAmCherry-KFERQ-NE (#102365, Addgene) to evaluate the activity of CMA. PAmCherry-KFERQ-NE was transduced into NPC by lentiviral transduction. To measure the activity of CMA, cells were photoactivated under a 405 nm LED light source 48 h after transduction and subjected to subsequent treatments. During this process, more than 90% of the cells were able to survive after photoactivation. Cells were fixed and co-stained with DAPI before microscopic imaging. Cell images were captured using a confocal fluorescence microscope (FV3000, Olympus, Japan). The activity of CMA was quantified by counting the number of red fluorescent spots per cell in at least three different fields of view under a 60x objective.

### Lysosome isolation

We isolated lysosomes from cell homogenates by differential centrifugation followed by density gradient centrifugation using the Lysosome Isolation Kit (#LYSISO1, Sigma-Aldrich, USA) following the manufacturer's protocol. Specifically, the homogenate was first centrifuged at 1 000 × *g* for 10 min at 4 °C to remove the nuclei. Next, the resulting supernatant was centrifuged at 20 000 × *g* for 20 min at the same temperature to pellet the lysosomes and other organelles. The supernatant was collected as the cytosolic fraction, while the pellet was the crude lysosomal fraction and resuspended in 1X extraction buffer. Following the manufacturer's instructions, we added OptiPrep and sucrose to construct a discontinuous density gradient medium and centrifuged at 150 000 × *g* for 4 h at 4 °C. The fractions were then collected, added with the appropriate amount of buffer, and centrifuged at 20 000 × *g* for 15 min to obtain the purified lysosomal pellet. Finally, we used an acid phosphatase assay kit (#CS0740, Sigma-Aldrich) to confirm the extraction of lysosomes.

### Nuclear and cytoplasmic separation

Nuclear and cytoplasmic separation was performed using a nuclear and cytoplasmic protein extraction kit (#P0028, Beyotime, China). According to the instructions, adherent cells were first collected and washed once with PBS. 200 µL of cytoplasmic protein extraction reagent A pre-added with PMSF was added to each 20 µL of the cell pellet. After vortexing, the mixture was placed on ice for 15 min. Then, cytoplasmic protein extraction reagent B was added. After vortexing, the mixture was centrifuged at 4 °C for 5 min. The supernatant was the extracted cytoplasmic protein. Nuclear protein extraction reagent was added to the remaining precipitate. After vortexing and mixing in an ice bath for 30 min, the mixture was centrifuged at 4 °C for 10 min. The supernatant was the extracted nuclear protein. All steps were performed on ice or at 4 °C to protect the protein from degradation.

### Glutamine assay

For glutamine measurement, glutamine in cell lysates was quantified using the Glutamine Assay Kit (Colorimetric) (#ab197011, Abcam, UK).

### Mitochondrial membrane potential and lysosomal staining

The mitochondrial red fluorescent probe (#C1035, Beyotime, China) was used to indicate the mitochondrial membrane potential according to the instructions. Specifically, when the cells were cultured to a certain density in the cell culture plate, the cell culture medium was removed, and the prepared Mito-Tracker Red CMXRos working solution was added and incubated at 37 °C for 20 min. After removing the staining working solution, fresh cell culture medium pre-incubated at 37 °C was added. For lysosomal staining, the kit (#40767ES50, YEASEN, Shanghai) was used, and the instructions were followed. The fluorescence intensity of the LysoSensor™ Green DND-189 probe increased in a pH-dependent manner (towards acid) as the acidification of the organelles increased. Observation was performed using a fluorescence microscope (Olympus, Japan). ImageJ was used to quantify the average fluorescence intensity of the cells.

### Statistical analysis

All data presented are the mean values and standard errors (SEM) obtained from multiple independent experiments (at least three times). The significant differences between the data were evaluated by a two-tailed Student’s t-test and one-way or two-way analysis of variance. Statistical analysis and graphs were prepared using GraphPad Prism 9 software. Different levels of significant differences are represented by the following symbols: *P <0.05, **P <0.01, ***P <0.001, ns indicates not significant.

## Supplementary information

SUPPLEMENTAL MATERIAL

## Data Availability

The data that support the findings of this study are available from the responding author upon reasonable request.
