# Supplementary material for: Chaperone-mediated autophagy directs a dual mechanism to balance premature senescence and senolysis to prevent intervertebral disc degeneration
Source: Bone Res. 2025 Jun 12;13:62. doi: 10.1038/s41413-025-00441-0 (PMC12162876; doi:10.1038/s41413-025-00441-0)
Supplement: Supplementary file 1 — SUPPLEMENTAL MATERIAL [file 41413_2025_441_MOESM1_ESM.docx]

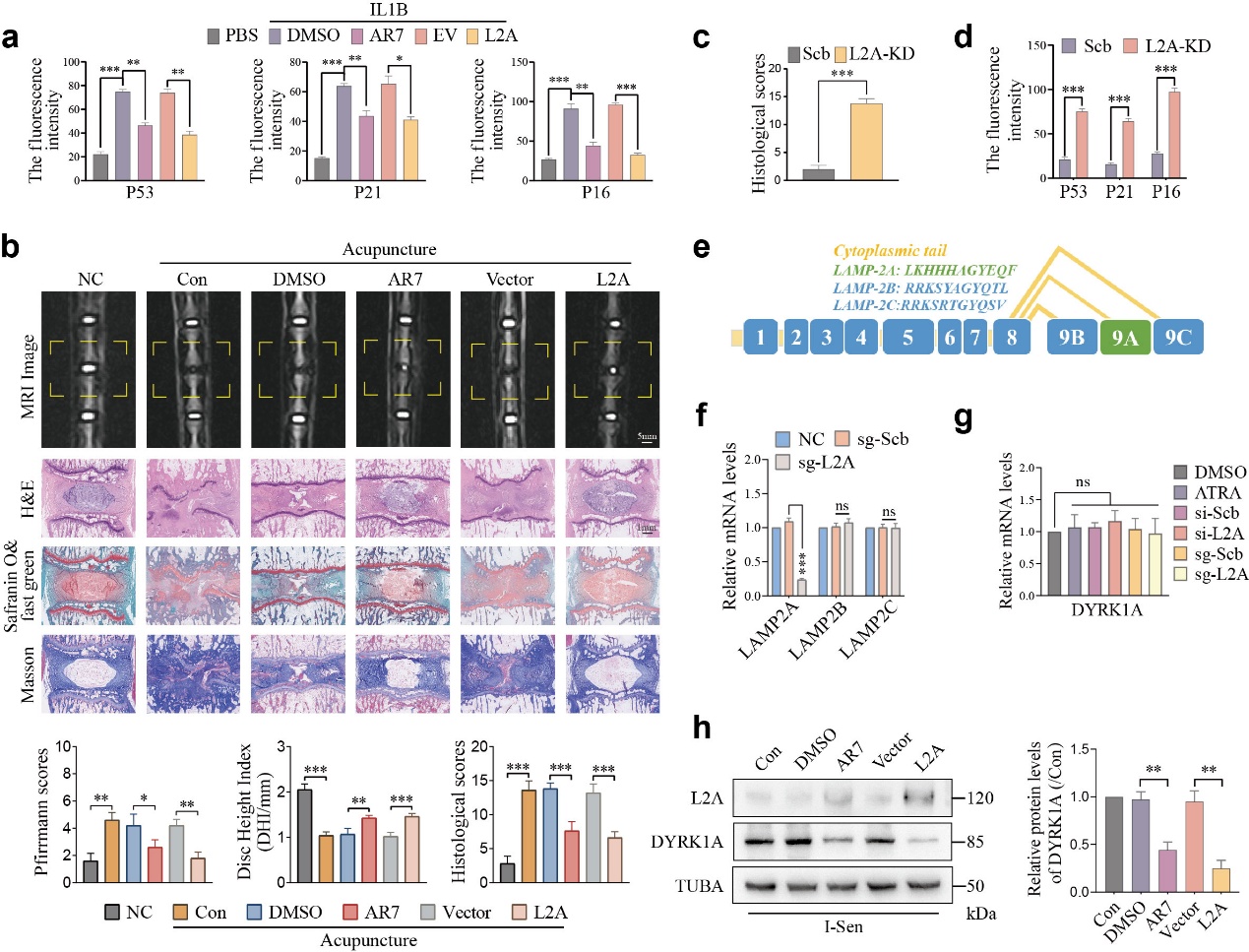


**Fig. S1. (a)** Quantification results of the average cell fluorescence intensity of P53, P21 and P16 in (Fig.1i). **(b)** MRI results and histological staining showing the effect of AR7 or L2A overexpression on acupuncture-induced IDD. The figure below shows the Pfirrmann scores, disc height index, and histological scores. **(c)** Histological scoring results of rat intervertebral disc after L2A-KD in (Fig. 1j). **(d)** The mean cellular fluorescence intensity of P53, P21 and P16 in (Fig.1m) after L2A-KD. **(e)** Schematic diagram of the gene sequence encoding LAMP2. The three isoforms of LAMP2 (A, B, and C) are derived from alternative splicing of exon 9. **(f)** RT-qPCR showing the mRNA levels of the other two Lamp2 isoforms (Lamp2b and Lamp2c) after knockout of L2A in NPC. **(g)** RT-qPCR showing the relative mRNA levels of DYRK1A in NPC treated with ATRA, si-L2A, or sg-L2A. **(h)** Western blot showing the protein levels of DYRK1A after AR7 treatment or L2A overexpression in I-Sen NPC. All graphs show the mean ± SEM of at least three independent experiments. */#p<0.05, **/##p<0.01, ***/###p<0.001, and ns indicates not significant.


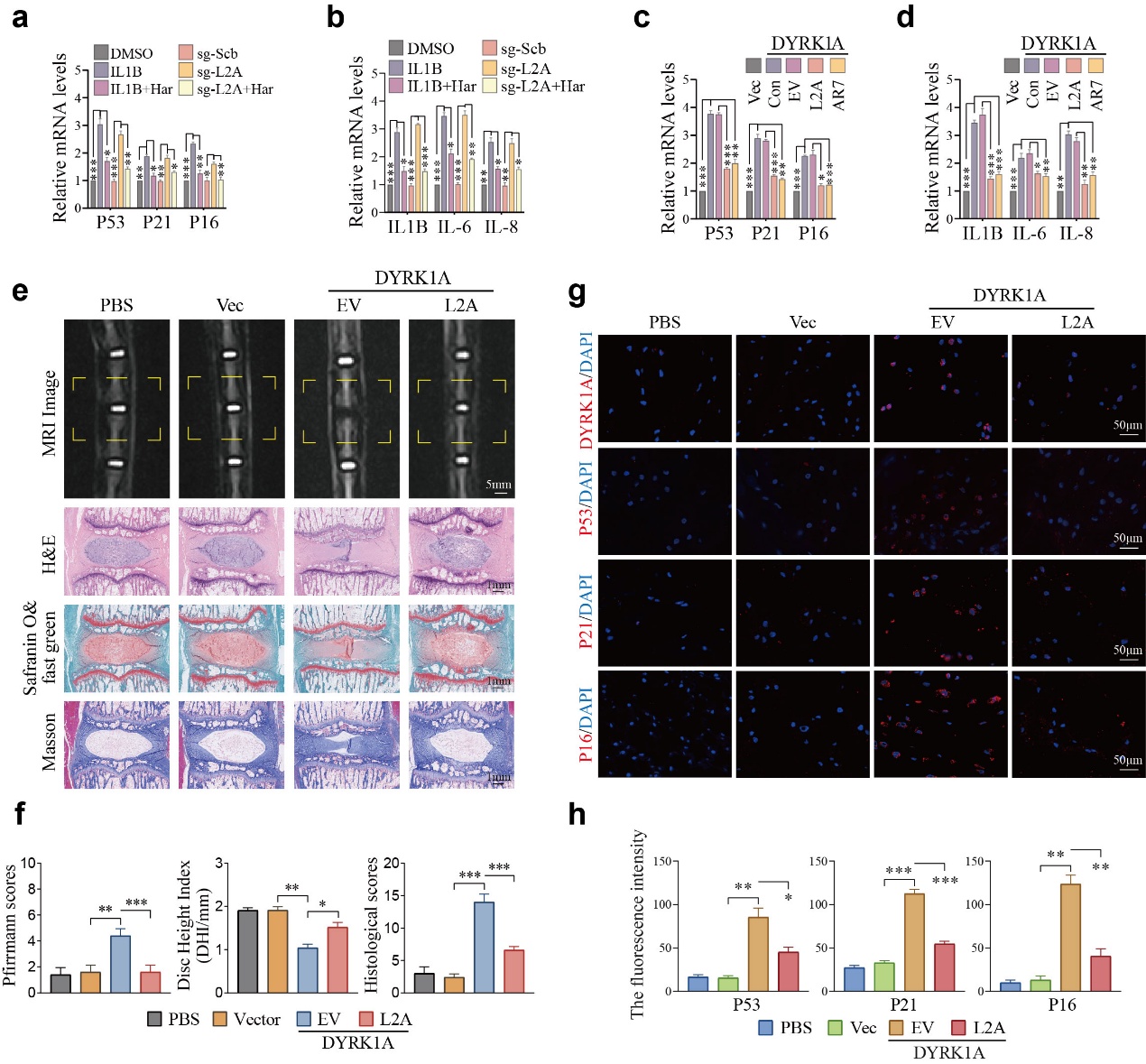


**Fig. S2. (a-b)** RT-qPCR showing the mRNA levels of senescence-related markers (P53, P21, and P16) and SASP inflammatory factors (IL1B, IL6, and IL-8) in NPC of each group. **(c-d)** RT-qPCR results show the relative mRNA levels of senescence markers (P53, P21, and P16) and SASP factors (IL1B, IL6, and IL-8) in NPC after DYRK1A overexpression or combined with CMA activation. **(e)** MRI results and histological staining showing the effect of DYRK1A overexpression or combined with L2A overexpression on rat IDD. **(f)** Effects of DYRK1A or combined with L2A overexpression on IDD as determined by Pfirrmann scores, disc height index, and histological scores. **(g-h)** Effects of DYRK1A or combined with L2A overexpression on the expression levels of senescence markers in rat intervertebral discs, including P53, P21, and P16. All graphs show the mean ± SEM of at least three independent experiments. */#p<0.05, **/##p<0.01, ***/###p<0.001.


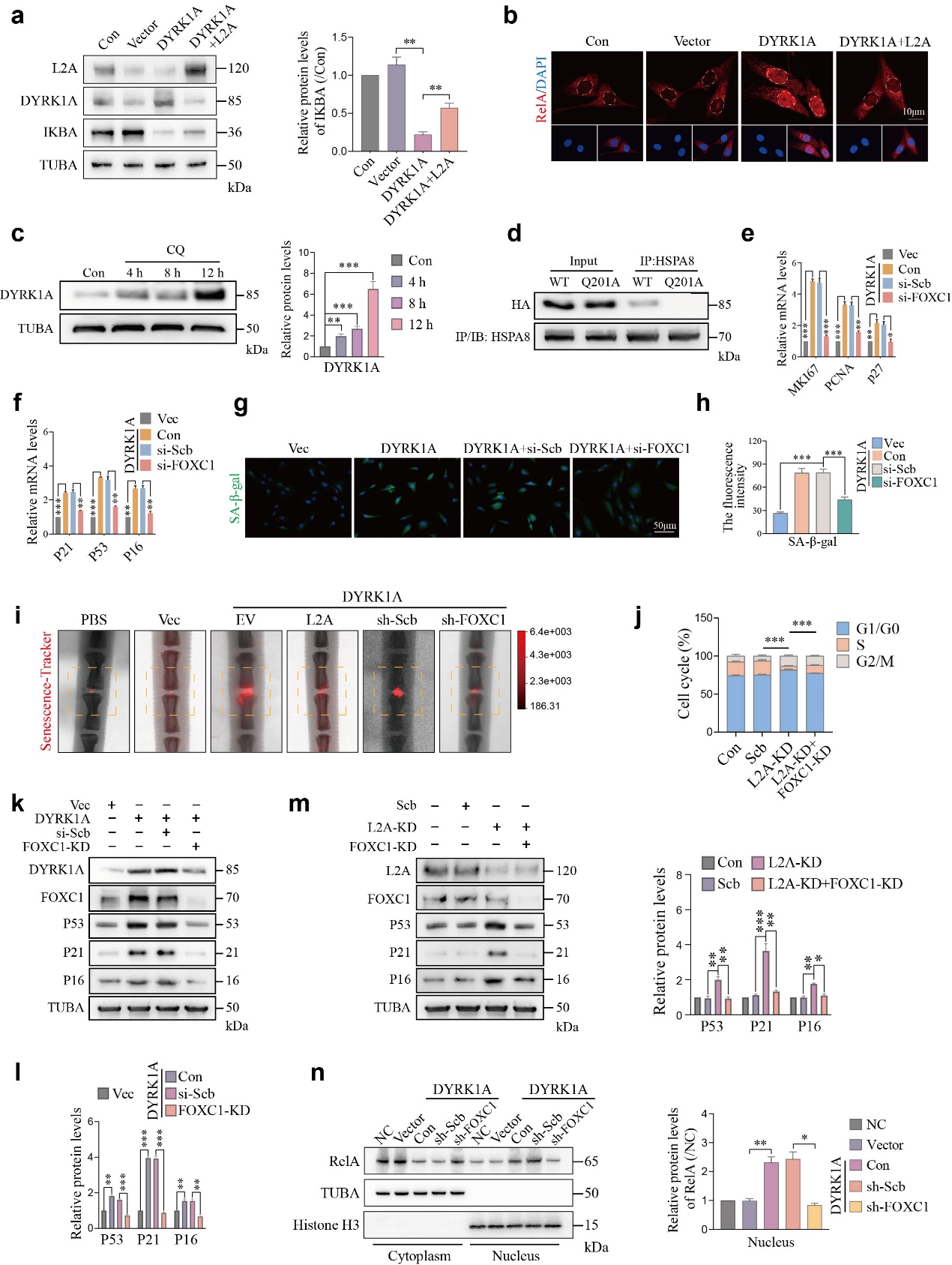


**Fig. S3. (a)** Western blot showing the effect of DYRK1A overexpression or combined with L2A overexpression on the expression levels of IKBA in NPC. **(b)** Immunofluorescence showing the effect of DYRK1A or combined with L2A overexpression on the nuclear translocation of RelA in NPC. **(c)** Western blot showing DYRK1A levels in NPC treated with CQ (20 μM) for different time periods (4 h, 8 h, and 12 h). **(d)** Co-immunoprecipitation shows the levels of HA bound to HSPA8 in NPC of WT and Q201A mutant groups. **(e-f)** RT-qPCR results show the changes in the mRNA levels of cell proliferation indicators (MKI67, PCNA, p27) and senescence-related proteins (P53, P21, P16) after DYRK1A overexpression or combined with si-FOXC1 transfection in NPC. **(g-h)** Fluorescent levels and quantification of SA-β-gal in NPC after DYRK1A overexpression or combined with FOXC1 knockdown. (**i)** The results of *in vivo* animal imaging based on SA-β-gal infrared fluorescent probes showing the effects of DYRK1A overexpression or combined with L2A overexpression or FOXC1 knockdown on rat disc senescence. **(j)** Flow cytometry results showing the effect of L2A knockdown or combined with FOXC1 knockdown on the cell cycle of NPC. **(k-l)** Western blot showing the effect of DRYK1A overexpression or combined with FOXC1-KD on NPC senescence. **(m)** Immunoblotting showing the effect of L2A knockdown or combined with FOXC1 knockdown on the protein levels of P53, P21, and P16 in NPC. **(n)** The results of immunoblotting experiments based on nuclear-cytoplasmic separation showed the effect of DYRK1A overexpression or combined with FOXC1 knockdown on RelA nuclear translocation. All graphs show the mean ± SEM of at least three independent experiments. */#p<0.05, **/##p<0.01, ***/###p<0.001.


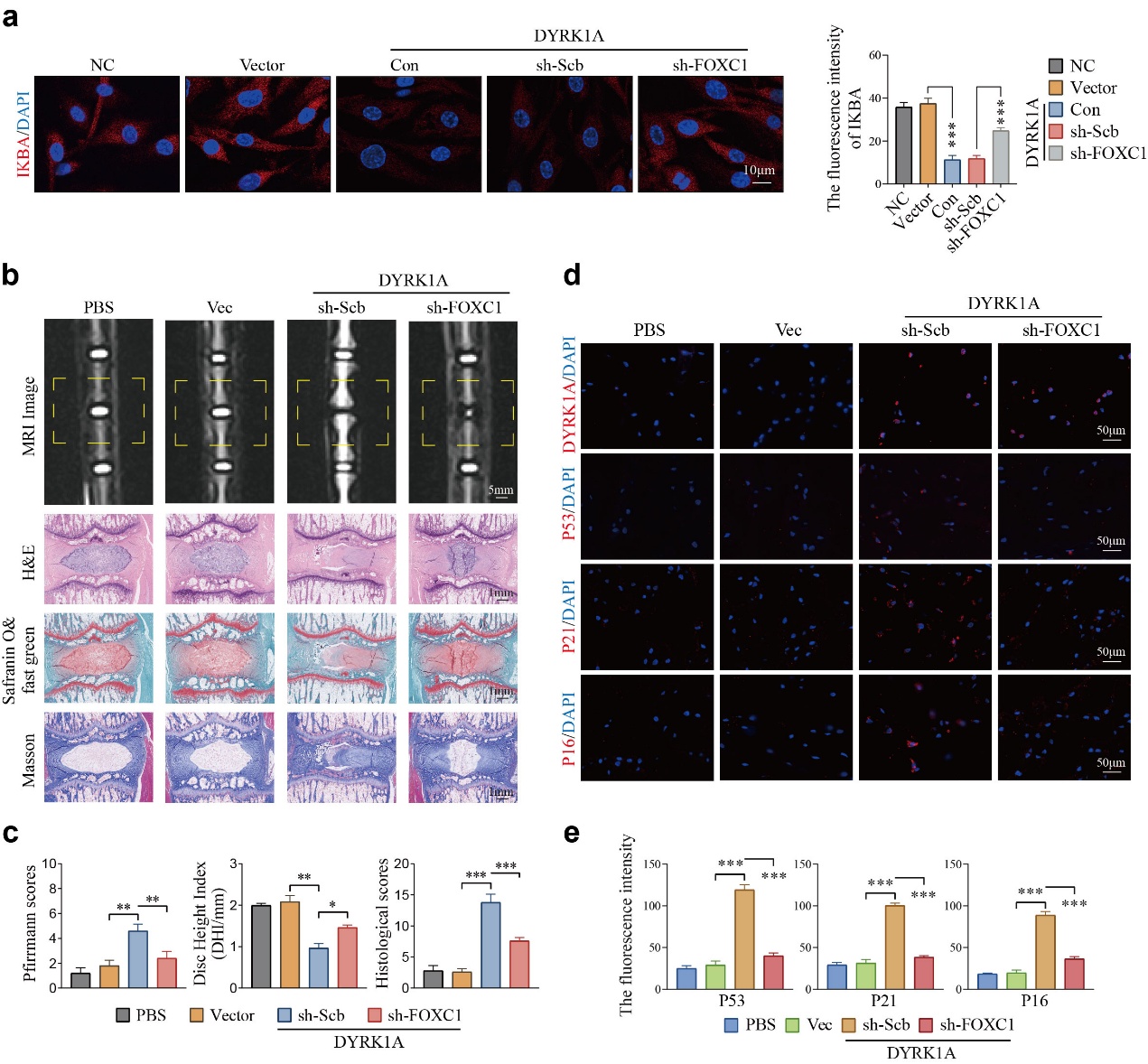


**Fig. S4. (a)** Immunofluorescence results showing the effect of DYRK1A overexpression or combined with FOXC1 knockdown on IKBA expression levels. **(b-c)** MRI results and histological staining showing the impact of DYRK1A overexpression alone or combined with sh-FOXC1 lentivirus injection on rat IDD. The rat IDD were assessed using the Pfirrmann scores, disc height index, and histological scores. **(d-e)** The influence of DYRK1A overexpression or combined with FOXC1 knockdown on the expression of P53, P21, and P16 in rat discs. All data are presented as the mean ± SEM from at least three independent experiments. */# p < 0.05, **/## p < 0.01, ***/### p < 0.001.


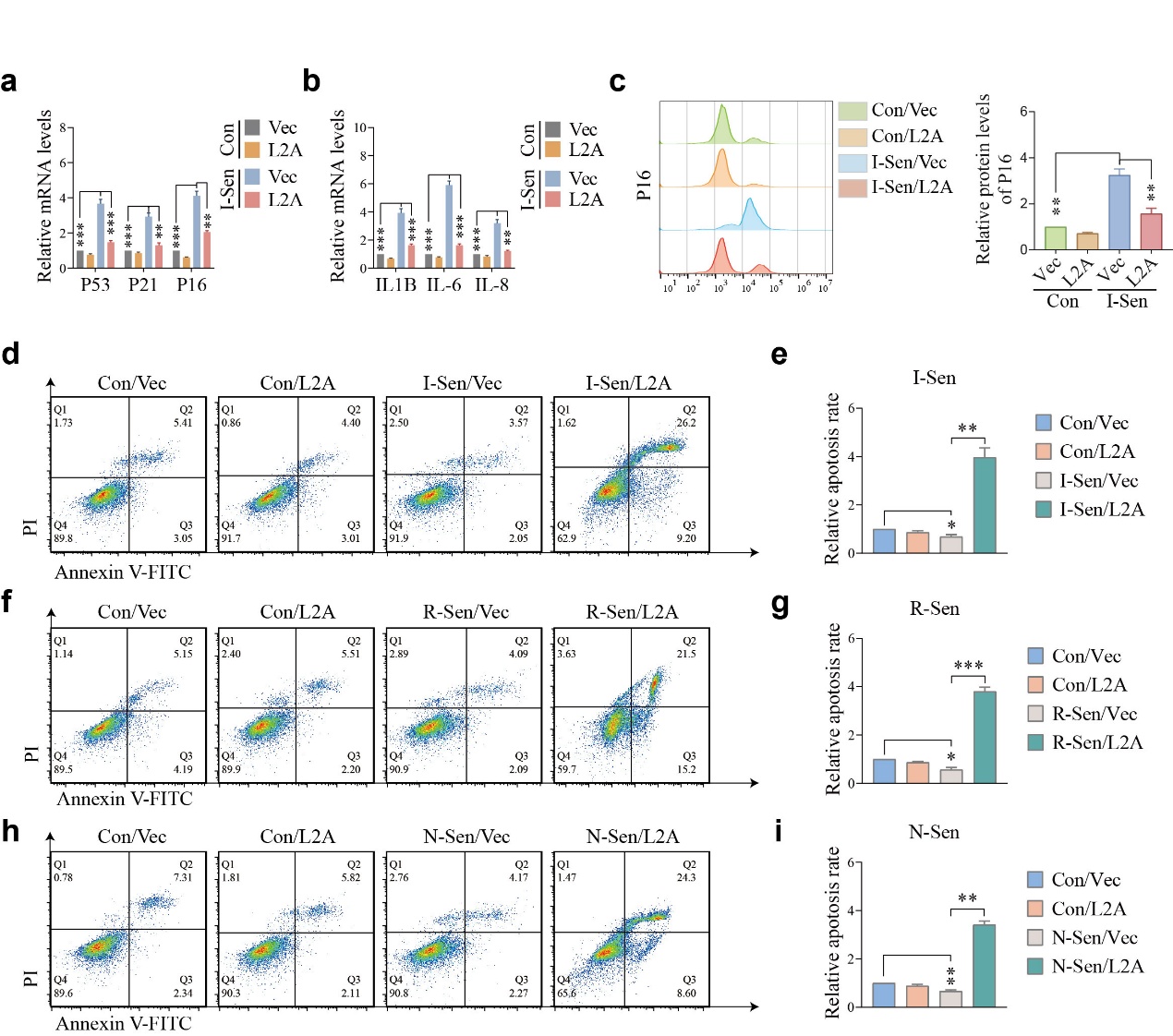


**Fig. S5. (a-b)** RT-qPCR results showing the effect of L2A overexpression on the mRNA levels of senescence-related markers (P53, P21, P16) and SASP inflammatory factors (IL1B, IL6, IL-8) in the control or I-Sen group. **(c)** The cell flow cytometry results show the effect of L2A overexpression on the levels of P16 in normal or I-Sen NPC. **(d-e)** Flow cytometry results showing the effect of L2A overexpression on apoptosis in normal or I-Sen NPC. **(f-g)** Flow cytometry results show the effect of L2A overexpression on apoptosis in normal or R-Sen NPC. **(h-i)** The effect of L2A overexpression on apoptosis in normal or N-Sen NPC. All graphs show the mean ± SEM of at least three independent experiments. */#p<0.05, **/##p<0.01, ***/###p<0.001.

**
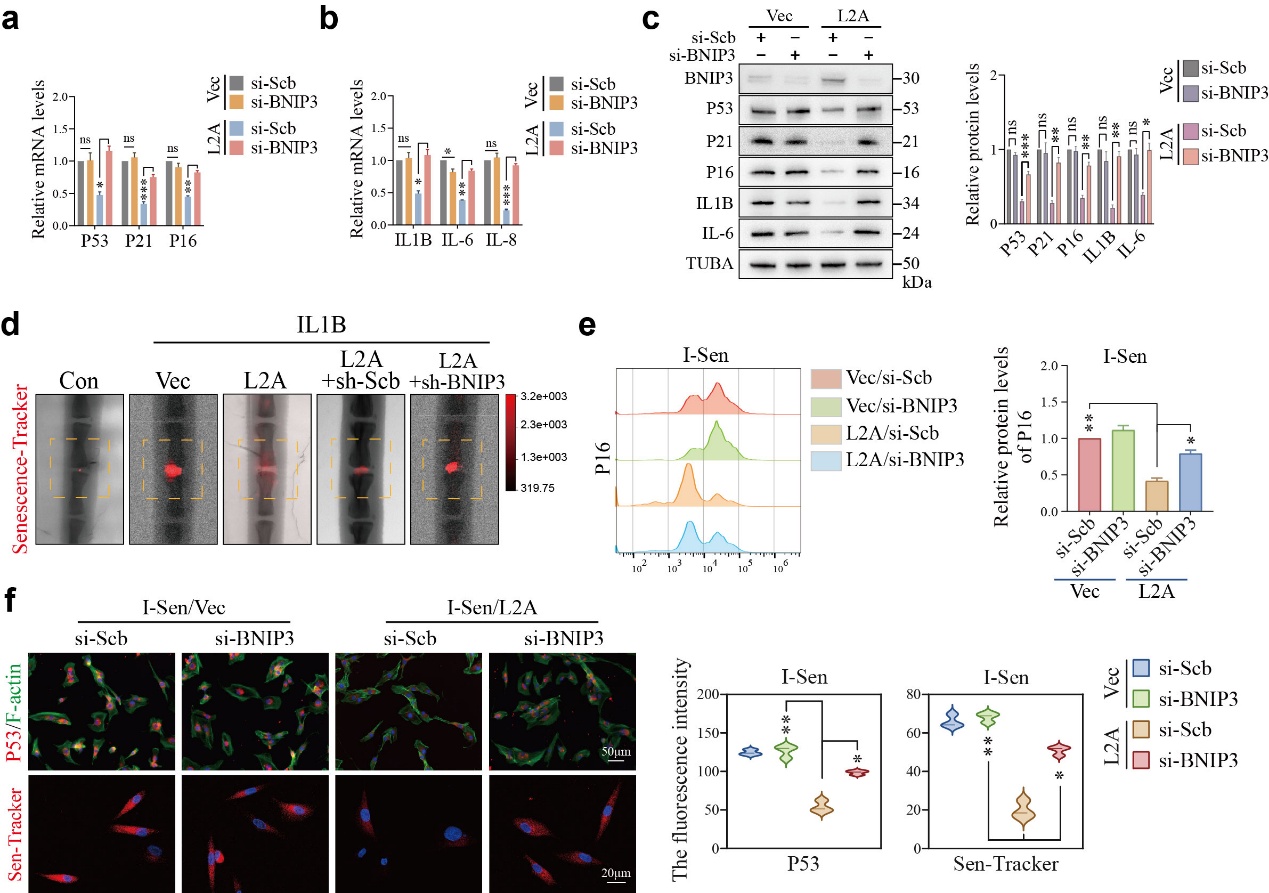
**

**Fig. S6. (a-b)** RT-qPCR showing the effect of L2A overexpression or combined with BNIP3 knockdown on the mRNA levels of senescence-related proteins (P53, P21, P16) and SASP inflammatory factors (IL1B, IL6, IL-8) in I-Sen NPC. **(c)** Immunoblotting showing the effect of L2A overexpression or combined with si-BNIP3 transfection on the levels of P53, P21, P16, IL1B, and IL6 in NPC of each group. **(d)** The *in vivo* animal imaging results based on SA-β-gal infrared fluorescent probe show the effects of IL1B or combined with L2A lentivirus, or with L2A and sh-BNIP3 lentivirus injection on rat disc senescence. **(e)** Flow cytometry results show the effect of L2A overexpression or combined with si-BNIP3 on P16 expression in I-Sen NPC. **(f)** Immunofluorescence showing the effect of L2A overexpression or combined with BNIP3 knockdown on P53 and senescence levels in I-Sen NPC. All graphs show the mean ± SEM of at least three independent experiments. */#p<0.05, **/##p<0.01, ***/###p<0.001, and ns indicates not significant.


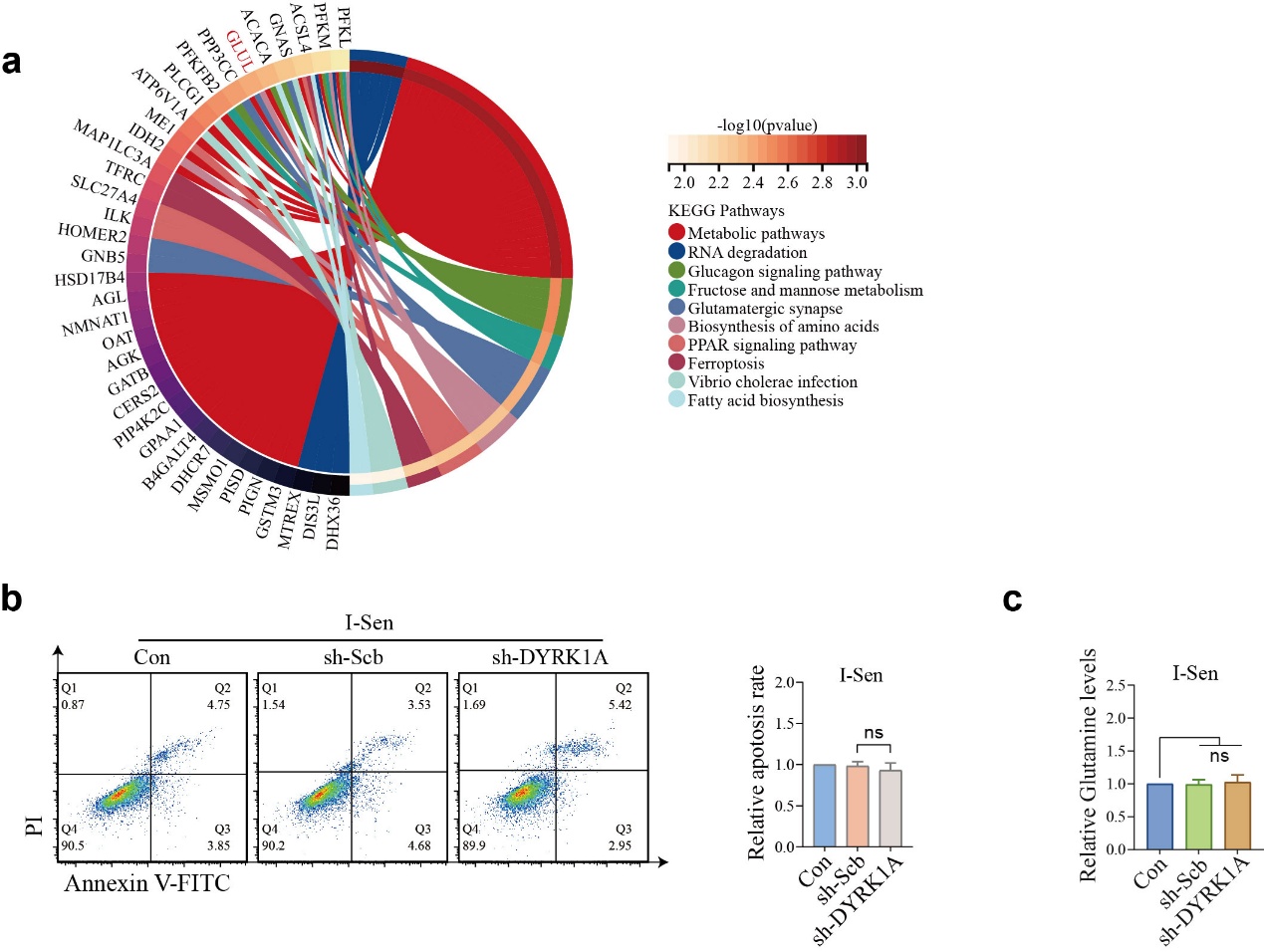


**Fig. S7. (a)** The results of proteomic data analysis after L2A knockout showing that CMA inhibition affects metabolic pathways of NPC and GLUL levels. **(b)** The cell flow cytometry analysis showing the effect of DYRK1A knockdown on apoptosis levels of I-Sen NPC. **(c)** Effect of sh-DYRK1A transfection on glutamine levels in I-Sen NPC. All graphs show the mean ± SEM of at least three independent experiments. Statistical significance was assessed by Student's t-test, and ns indicates not significant.


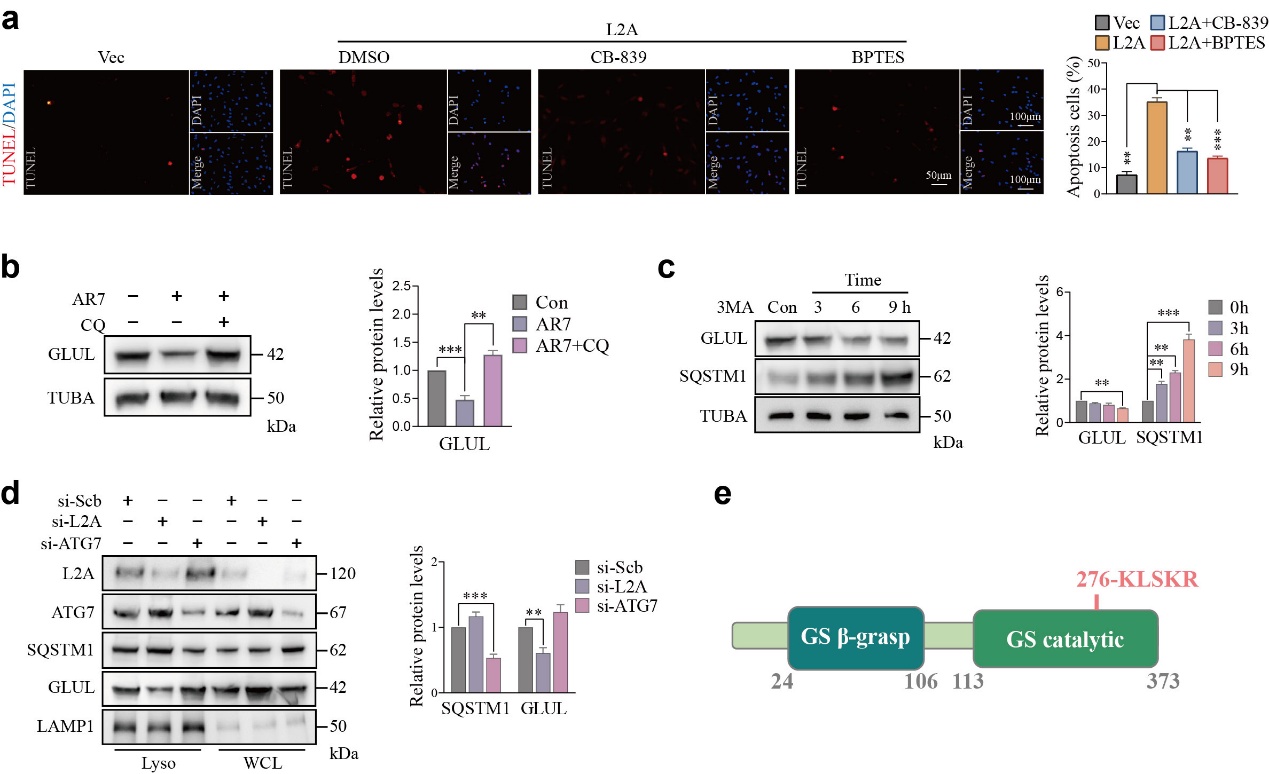


**Fig. S8. (a)** TUNEL staining showing the effect of L2A overexpression or combined with CB-839 or BPTES on apoptosis of I-Sen NPC. **(b)** Western blot showing the level of GLUL in NPC after treatment with AR7 or CQ. **(c)** Immunoblotting showing the protein level of GLUL and SQSTM1 in NPC after treated with 3MA (5 mM) for different time periods. **(d)** Immunoblotting shows the protein level of SQSTM1 and GLUL in lysosomes of NPC after L2A knockdown or ATG7 knockdown. **(e)** Schematic diagram of the protein structure sequence of GLUL. All graphs show the mean ± SEM of at least three independent experiments. */#p<0.05, **/##p<0.01, ***/###p<0.001.
